# Supplementary material for: Computer‐aided biochemical programming of synthetic microreactors as diagnostic devices
Source: Mol Syst Biol. 2018 Apr 26;14(4):e7845. doi: 10.15252/msb.20177845 (PMC5917673; doi:10.15252/msb.20177845)

Appendix for:

Computer-aided biochemical programming of synthetic microreactors as diagnostic devices

This PDF File includes:

I. Appendix materials and methods.

1. *Microfluidics and protosensor preparation and characterization*
2. *In silico modeling, simulation, and computed output signals*
3. *Microscopic size dispersion measurements*
4. *Supplementary references*

II. Appendix Figures and Tables.

Appendix Table S1*. Stock solutions and concentrations used in this study*

Appendix Table S2*. Enzymatic and kinetic parameters of biochemical species used in this study*

Appendix Figure S1*. Example of automated biochemical implementation of user defined enzymatic Boolean logic gate from natural metabolic networks*

Appendix Figure S2*. Topology of biochemical circuits with kinetic parameters that were designed and used in this study*

Appendix Figure S3*. Multisubstrate enzymatic mechanisms describing biochemical reactions and corresponding HSIM models equations*

Appendix Figure S4*. Experimental calibration curves used to compute output signals*

Appendix Figure S5*. Detailed experimental kinetic characterization of synthetic biochemical circuits in vitro*

Appendix Figure S6*. Detailed experimental logic characterization of synthetic biochemical circuits in vitro*

Appendix Figure S7*. Microfluidic chip used in this study to generate protosensors*

Appendix Figure S8*. Double emulsion template for protosensor directed self-assembly*

Appendix Figure S9*. Environmental Scanning Electron Microscopy (ESEM) photomicrograph of protosensors*

Appendix Figure S10*. Time-lapse photomicrograph of protosensors fabrication within microchannels*

Appendix Figure S11*. UPLC-Mass spectrometry experiments to assay enzyme encapsulation in protosensors*

Appendix Figure S12*. Mapping satisfaction degree landscape for (A) GluOne, (B) LacOH and (C) GluNOx biochemical circuits in protosensors*

Appendix Figure S13*. Experimental validation of GluONe Out1 using confocal microscopy*

Appendix Figure S14. *Detailed experimental kinetic characterization of synthetic biochemical circuits in protosensors operating in urine*

Appendix Figure S15*. Experimental truth tables of protosensors operating in PBS*

III. HSIM code for models used in this study

1. GluONe batch mode

2. GluONe protosensor mode

3. LacOH batch mode

4. LacOH protosensor mode

5. GluNOx batch mode

6. GluNOx protosensor mode

IV. Example of BIOCHAM code for biochemical programming

Supplementary files attached:

1. Synthetic biochemical circuits in SBML format:

Computer Code EV[1].xml

Computer Code EV[2].xml

Computer Code EV[3].xml

Computer Code EV[4].xml

2. Biocham Notebooks

Computer Code EV[5].bc

Computer Code EV[6].bc

Computer Code EV[7].bc

3. Microfluidic protosensor fabrication

Movie EV1.mp4

I. Appendix materials and methods.

1. Microfluidics and protosensor preparation and characterization

PDMS microfluidic chips were designed and prototyped using AutoCAD software and fabrication was carried out by the Stanford University microfluidic foundry. 20 Gauge holes were punched in the PDMS chip, allowing the use of customized made stainless steel adapters (New England Small Tube) for PTFE tubing connections (1/16 OD, 0.8 mm ID). The flow in microfluidic channel was controlled via displacement driven flow using Cetoni neMESYS syringe pumps equipped with high precision glass syringes. We found strong dependence of protosensors yields of production on flow rates, that were kept at 1/0.4/0.4 µl/min (A/B/C) to achieve best encapsulation efficiency. Movies taken with an ultrafast camera (Phantom v7.3) at 20 000 FPS allows us to estimate around ~1500 Hz the frequency of protosensor generation at these flow rates (the time-lapse corresponding to the generation of one protosensor shown in Appendix Figure S10 corresponds to ~500 µs).

We introduced in our microfluidic design a device previously described, known as the staggered herringbone mixer (SHM) (Williams et al., 2008). Wet enables efficient passive, chaotic mixing between different solutions under Stokes-flow regime. We introduced two times more cycles (i.e. 10), as we calculated that ~5 cycles were sufficient to efficient mixing, according to the equations provided in Williams et al.. We integrated this device in our designs to achieve full mixing of the two C channels just before encapsulation, in order to ensure homogeneous internal content, precise stoichiometry, and efficient encapsulation, which could have been affected by laminar biochemical gradients and spatial anisotropy of concentrations. Synthetic biochemical circuits can then be spontaneously assembled just before encapsulation, by that mean standardizing the encapsulation mechanism and reducing its dependency on the nature of biochemical materials. Moreover, this design allows for fine tuning on stoichiometry via control on the input flow rates, which proved practical to test different parameters for straightforward prototyping of protosensors.

Our strategy relies on a microfluidic flow-focusing droplet generation design that generates water-in-oil-in-water (W–O–W: Biochemical circuit in PBS - Phospholipid in Oleic acid - Buffer A) double emulsions. Double emulsion templates are generated in described flow-focusing channel geometries. DPPC phospholipid membranes then self-assemble during a controlled solvent extraction process (Oleic acid is extracted by methanol present in buffer A). The rational we use for choosing DPPC concentration in oleic acid is adapted from Teh *et al.*. Briefly, oleic acid solution was composed of 1.1mM DPPC. This concentration was chosen so that there would be a sufficient number of phospholipids to form a lipid bilayer around a 10µm diameter vesicle. The average area per molecule of DPPC in a bilayer membrane is estimated around a value of 0.64 nm²(Nagle & Tristram-Nagle, 2000). Considering that a 10µm liposome would have a lipid area of $3.14.10^{-10}$ m², at least $8.15.10^{-16}$ moles of DPPC would be required to compose the full lipid bilayer of a liposome. Assuming the maximum thickness of oleic acid contained by the primary double emulsion to be 5µm, we can calculate the volume of oleic acid to be 3.665 pL. A 1.33 mM DPPC concentration would be sufficient, as is it 5 times the amount of phospholipid needed to form a lipid bilayer around the vesicle. In addition, we briefly investigated DOPC, DMPC and DPPC phospholipids for protosensor fabrication, and found that DPPC achieved better apparent stability and superior production yields.

In order to achieve selective biomarker input entry and matter/information exchange between protosensor content and exterior medium, we capitalized on passive pore forming bacterial protein α-hemolysin. α-hemolysin pores have several properties that identifies it as a robust transmembrane channel suitable for biosensing applications. α-hemolysin pores are self -assembled in the membrane and do not require specific assembly conditions, they are stable over a wide range of pH and temperature and are open in normal conditions. The transmembrane pore of α-hemolysin operates the delivery of ions and small organic compounds such as sugars, metabolites or nucleotides in a selective way through the walls of synthetic lipid vesicles (Aksimentiev et al., 2005) with a passive diffusion rate of $5.5 \pm 1.5 \times 10^{-4}\ s^{-1}$ as previously measured (Watanabe et al., 2014).

The output channel of the microfluidic chip containing newly formed protosensors was connected with PTFE tubing to a collection vial containing buffer A kept on ice. After 5 hours of fabrication which also allows methanol to evaporate, we obtained ~1 ml of protosensor (by encapsulated volume). The final solution containing protosensors corresponding to a ~1:5 final dilution in buffer A (storage buffer) could then be stored at 4°C for a maximum time of 1 week before further use.

For measurements, hemolysin treatment of protosensor was performed 15 minutes prior to induction. Hemolysin was added for a final concentration of 1 µM (33 ug/ml). Protosensors solutions were then back diluted at a 1:1 proportion into the sample to test (e.g. Urine). Induction was carried out under slow agitation at 25°C. All fluorescence and absorbance measurements were performed on a synergy H1 plate reader, in 100µl p96 microwells.

2. In silico design, modeling, simulation, and computed output signals

The capacity to rationally design biological systems to achieve programmed biosensing and user-defined decision-making algorithm and bioactuation requires precise tools within the scope of a systematic approach. Therefore, we first developed an *in silico* framework supporting the design of synthetic biochemical circuits from the bottom-up assembly of biological parts (**Figure 1**). This computer aided framework involves the following steps:

(i) Design of abstract programs with respect to Boolean logic and molecular input/output specifications according to (medical) algorithm of interest. At this step, one can formalize the temporal logic properties of a synthetic biochemical system regarding expected reference behavior, expressed by a qualitative and/or quantitative temporal logic formula (Rizk et al., 2009). This comprises the specification of parameters relative to the initial state (i.e. pathological biomarker concentration thresholds for instance).

(ii) Implementation of previously defined algorithms using molecular biochemical circuitry: identification of suitable enzymes and metabolites within a network topology obtained from databases and literature to implement molecular Boolean logic operations. Composable and kinetically favorable components are chosen at this step to minimize modes of failure. This process can be automated using recently developed computer aided extraction of biochemical parts, and Boolean logic gates from metabolic networks of living organisms using NetGate, a part of the Silicell Maker software (Mehta et al., 2015). Experimental characterization of biochemical modules can help this process. Silicell Maker software if used for user friendly design and mapping of interaction network and to assign reaction rules (with or without kinetic expressions), concentration parameters, as well as spatial parameters such as volume, location of species, and permeability coefficients of compartments. Silicell Maker is then used for automated generation of HSIM and BIOCHAM code for the model (specification of the initial state, definitions of numerical parameters, compartments volume, invariants, events, declarations of molecular species and locations, specification of the system's behavior).

(iii) Stochastic simulation (SSA) is first performed within the HSIM software to verify kinetically and functionally favorable circuits, predict the overall behavior, estimate the functioning and manually explore the design space to identify suitable parameter configurations to be refined.

(iv) BIOCHAM simulations (ODE solver) are then carried out to compute validity domains of specified behavior for the system, perform sensitivity analysis to identify sensitive parameters that can then be iteratively optimized, and measure robustness relative to the variation of specific parameters. Models are thus evaluated with respect to temporal logic specifications. Computing a landscape of satisfaction relative to sensitive parameters enables to visualize and identify suitable parameter space satisfying specified behavior, which can be used to select robust parameters for experimental *in vitro* implementation (i.e. concentration of species for instance, kinetic parameters, initial value or control parameters). One can use automate the search, using CMAES methodology (Covariance Matrix Adaptation Evolution Strategy) (Hansen & Ostermeier, 2001) integrated in BIOCHAM for those parameter values that satisfy a given set of quantitative temporal properties.

(v) Once the parameters satisfying user-defined system specifications have been found, HSIM stochastic simulator can be used to validate and finely map the complete transfer functions of the protosensors. The systems can then be experimentally implemented and their functioning assessed *in vitro*. Iterations in the design process can occur at each step.

NetGate and NetBuild

Since our goal is to build complex biochemical logic circuits using enzymatic reactions that take place simultaneously in the same environment (*i.e.* a microreactor), it is important to extract molecular species that are part of the same metabolic network *in vivo* in order to minimize potential failure modes. The program NetGate and NetBuild available in the Silicell Maker software, define biochemical logic gates by their truth table, the set of molecular species representing input substrates, output products, and enzyme. As described in details in Bouffard et al.[71] these programs use the important following definitions:

1. A metabolic network is a set of interconnected reactions involving metabolites (substrates or products) and enzymes (catalysts or modulators). They form a dense and usually strongly connected network.
2. Tied reactions: two reactions are tied if they share at least one common molecular species.
3. A logic gate is an abstract construction with at least one input and one output. A truth table is associated to the gate; The truth table gives the value of each output for each possible boolean pattern of the inputs. The set of logic gates NetGate is searching for are described by their truth table in a parameter file.
4. An implementation of a logic gate is a subnetwork of the input metabolic network where the inputs and output are identified. The number of inputs of the subnetwork may exceed the number of inputs of the gate.If the value of one of these extra-input does not change the behavior of the gate, this input is left free and will be ignored. Conversely, if any variation of the value of an extra-input changes the behavior of the gate, then a fixed boolean value is assigned to this input in order to get the correct truth table for the gate.

NetGate and NetBuild take as inputs (i) a SBML file describing a metabolic network such as the one of **Appendix Figure S1A**, but can consist of significantly larger networks such as the ones that can be found in the MetaCyc Metabolic Pathway Database and (ii) a list of truth tables corresponding to the logic gates that are to be searched in the metabolic network. First, all the possible implementations of the logic gates are enumerated; Then, these implementations are checked against the given list of truth tables and the gates found are sorted and output. The gates implementations are searched in subnetworks extracted from the original metabolic network. These subnetworks are built starting from one reaction of the original network, the seed, then adding successively other reactions that are tied to this seed. To get all the subnetworks, this process is repeated starting from all the reactions of the original metabolic network. Then, for each of the given gate description, all the possible implementations are searched within each subnetwork. All the mappings of the inputs of the gate to the inputs of the subnetwork are

successively checked to see if all the lines of truth table of the gate description can be obtained. The in-depth description of the algorithm can be found in Bouffard et al.

HSIM models

For all simulations, we used a protosensor diameter of 10µm. To generate heat maps, we ran 5 simulations for each point in order to average for stochastic behavior.

Modeling protosensor permeability:

For the purpose of this study, we implemented in HSIM the diffusion rate *dn/dt* (in mol/s) of input metabolites from the medium to the inside of protosensors. This is driven by passive diffusion, given by a modification of *Fick's law*, which states that the diffusion rate across the membrane of width *x* is directly proportional to the *permeability coefficient P,* to the difference in solution concentrations $C_1^{aq} - C_2^{aq}$, and to the area *A* of the protosensor, or

$$\frac{dn}{dt} = PA(\frac{C_{\text{exterior}} - C_{\text{protosensor}}}{dx})$$

With for any molecule, the value of *P*, and thus its rate of passive diffusion, is proportional to its partition coefficient *K*:

$$P = \frac{kD}{x}$$

Where *D* is the diffusion coefficient of the substance within the membrane and *x* is the membrane thickness. These experimental parameters can be easily found in the literature for a wide panel of molecules diffusing across phospholipid bilayers. HSIM supports the introduction of permeability coefficient *P* ($m.s^{-1}$). For this study, we used a *P* value for ethanol and acetone diffusing passively across DPPC bilayer of around 0.01 $m.s^{-1}$. Phospholipid bilayers being naturally impermeable to other organic solutes, we introduced staphyloccus α-hemolysin pore forming protein in the membranes of protosensors, in order to allow passive diffusion of input biomarkers metabolites. The diffusion coefficient has been widely measured, and according to a recent measurement by Wanatabe *et al.* is estimated around $5.1.10^{-11}$ $m^2.s^{-1}$, which is interestingly only ~10 time smaller than in free aqueous solution. Considering the DPPC bilayer to be 3.2 nm wide (Stillwell, 2013), on can calculate the permeability coefficient, which gives us $1.6.10^{-2}$ $m.s^{-1}$. Assuming a hemolysin pore radius of 0.7 nm, and an overall protein radius of 4 nm, we can estimate that at membrane saturation with 1uM hemolysin ~3% of protosensors surface would be covered by hemolysin pores, which would then correspond to a permeability coefficient value of $0.5.10^{-3}$ $m.s^{-1}$. Importantly, we found that modeling the leaking of NAD+ metabolite through hemolysin pores, which is integrated in the biochemical circuits, did not seem to affect the functioning of the protosensors. In our experimental set up, before induction, the NAD+ concentration of the storage solution is at equilibrium with the interior of protosensors, and the 1:1 dilution with the medium to test does not induce a significant decrease in concentration in comparison to the kinetics of the enzymatic processes.

BIOCHAM models

Validity domains were computed to extract concentration thresholds (N and R) at steady state (T) satisfying temporal logic specifications. Starting with models containing non-optimized initial concentration parameters which did not necessarily complied to temporal logic specifications, we first performed sensitivity analysis on concentration parameters with a specified logic formula corresponding to requested systems behavior and desired input/output levels and thresholds. This permitted us to identify the two most sensitive concentration parameters of the systems, which we then used to visualize the design space through comprehensive map of configurations satisfying specifications. We then conducted an automated parameter search according to the stochastic optimization method CMAES implemented in BIOCHAM (covariance matrix adaptive evolution strategy (Hansen & Ostermeier). For instance, for the model GluNOx (**Computer Code EV7)**, this process would require the following commands in BIOCHAM (complete code can be found in **supplementary files** (**Computer Code EV5, EV6 and EV7**) and section **IV.** of **Appendix**):

```
% Trace analysis: extraction of thresholds (N and R) and switch time (T) at steady state (FG)
% Temporal specification of output concentration thresholds at steady state
% This equation corresponds to the expected behavior for inputs 1-1 (Glucose - NOx)

In [121]: validity_domain(F(G((Time>T) & (N > [NADH]) & ([DAFF] > R)))).

Simulation time: 6.252s
T < 20.0357, N > 14376.6, R < 2.11359e+06
Time elapsed : 124 ms

% LOCAL SENSITIVITY INDICES of all optimizable parameters with 0.5 variations
% The following equations correspond to the expected behavior for inputs 0-0 (Glucose - NOx), inputs 1-0 (Glucose - NOx), inputs 0-1 (Glucose - NOx) and
inputs % 1-1 (Glucose - NOx) respectively.

In [122]: sensitivity([g,a,b],[0.5,0.5,0.5],F(G((N > [NADH]) & (R > [DAFF]))), [N,R], [1000,1000], 300).

g=9.46014e+08 violation 4.99003e+07 (0.16s)
 g=3.15338e+08 violation 4.95135e+07 (0.15s)
Sensitivity[g] 1.22595e-20

 a=5.44431e+07 violation 4.99007e+07 (0.16s)
 a=1.81477e+07 violation 4.87336e+07 (0.13s)
Sensitivity[a] 1.1515e-19

 b=3.97326e+07 violation 4.94338e+07 (0.16s)
 b=1.32442e+07 violation 4.96738e+07 (0.14s)
Sensitivity[b] 4.77609e-21
```

```
In [123]: sensitivity([g,a,b],[0.5,0.5,0.5],F(G(([NADH] > N) & (R > [DAFF]))), [N,R], [1e7,1000], 300).
```

```
g=9.46014e+08 violation 5.08918e+07 (0.16s)
 g=3.15338e+08 violation 5.05125e+07 (0.15s)
Sensitivity[g] 1.08854e-20

 a=5.44431e+07 violation 5.08922e+07 (0.16s)
 a=1.81477e+07 violation 4.97483e+07 (0.13s)
Sensitivity[a] 1.02051e-19

 b=3.97326e+07 violation 5.04346e+07 (0.16s)
 b=1.32442e+07 violation 5.0669e+07 (0.14s)
Sensitivity[b] 4.20531e-21
```

```
In [124]: sensitivity([g,a,b],[0.5,0.5,0.5],F(G((N > [NADH]) & (R > [DAFF]))), [N,R], [1000,1000], 300).
```

```
g=9.46014e+08 violation 4.99003e+07 (0.16s)
 g=3.15338e+08 violation 4.95135e+07 (0.15s)
Sensitivity[g] 1.22595e-20

 a=5.44431e+07 violation 4.99007e+07 (0.16s)
 a=1.81477e+07 violation 4.87336e+07 (0.13s)
Sensitivity[a] 1.1515e-19

 b=3.97326e+07 violation 4.94338e+07 (0.16s)
 b=1.32442e+07 violation 4.96738e+07 (0.14s)
Sensitivity[b] 4.77609e-21
```

```
In [125]: sensitivity([g,a,b],[0.5,0.5,0.5],F(G((N > [NADH]) & ([DAFF] > R))), [N,R], [100000,1e8], 300).
```

```
g=9.46014e+08 violation 5.00987e+07 (0.15s)
 g=3.15338e+08 violation 5.04855e+07 (0.14s)
Sensitivity[g] 1.16988e-20

 a=5.44431e+07 violation 5.00983e+07 (0.16s)
 a=1.81477e+07 violation 5.12654e+07 (0.13s)
Sensitivity[a] 1.03239e-19

 b=3.97326e+07 violation 5.05652e+07 (0.16s)
 b=1.32442e+07 violation 5.03252e+07 (0.14s)
Sensitivity[b] 4.4473e-21
```

% VISUALIZATION of most sensitive parameters landscape, a and g, as determined by the previous calculations.

```
In [125]: landscape([g,a],[(0,1000000000),(0,100000000)],F(G((N > [NADH]) & ([DAFF] > R))), [N,R], [3500,50000000], 10, 300, landG1DHADH).
```

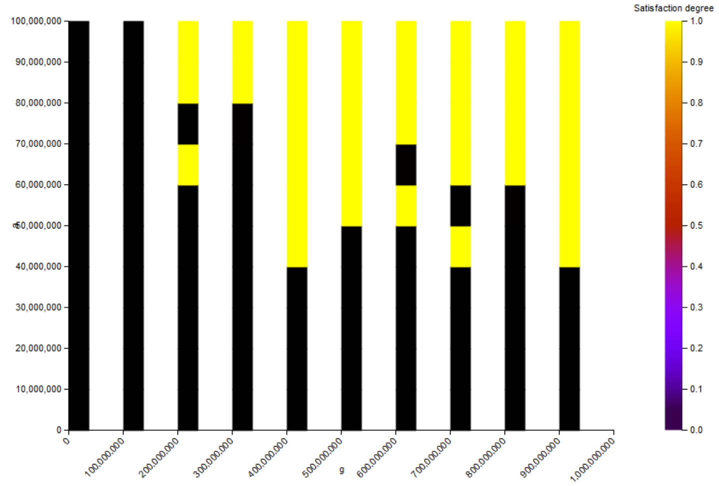

% PARAMETER SEARCH

```
In [127]: search_parameters_cmaes([g,a,b],[(0,1000000000),(0,100000000),(0,100000000)],F(G((N > [NADH]) & ([DAFF] > R))), [N,R], [3500,50000000],300).
```

```
## Exit function optimize (Thu Nov 16 09:45:15 2017) ##
Best parameter coefs : 1.04272 1.15752 0.957613
Found Parameters :
parameter(g,657619411).
parameter(a,42012496.1).
parameter(b,25365634.1).
Time elapsed 219.46 s
```

All analysis and *in silico* modeling were performed according to input concentration parameters corresponding to pathological threshold values. The following pathological threshold for input biomarkers were rationally specified according to clinical requirement, and used for calculations and parameter optimization:

Ketones>17 µM (10mg/dl, pathological if >0)
Glucose>1.39 mM (25mg/dl, pathological threshold)
Lactate>10 µM (pathological if >0)
EtOH>17.4 µM (80mg/dl, equivalent to DUI)
NOx>1000 µM

Multisubstrate enzymatic mechanisms implementation in Silicell Maker and HSIM:

In this study, the three enzymatic mechanisms depicted in **Appendix Figure S3** were implemented in HSIM in order to account for reaction kinetics: Ordered sequential bi-bi mechanism, Ping Pong bi-bi mechanism, and random sequential bi-bi mechanism.

Translation of probability from HSIM stochastic simulator to mass action rates for BIOCHAM ODE solver:

HSIM manages molecules in terms of copy number, and not in concentration terms, as it takes into account compartments volumes. BIOCHAM uses concentrations, where Mass action rate factors intrinsically integrate the volume parameter for a monomolecular reaction, in BIOCHAM as K is in volume/time and [A] in copy number/volume, K*[A] is the number of reactions that will happen per units of time. Likewise, HSIM integrate a constant time step in its probabilities. Similarly, for a bimolecular reaction, in BIOCHAM K is in volume²/time and [A] and [B] in copy number/volume, which gives us a number of reaction events per units of time. HSIM probabilities have thus to be translated into BIOCHAM Mass action rates. Mass action rate (MA) used in the BIOCHAM ODE solver can be related to HSIM stochastic simulator probabilities (P) according to:

$(MA) = \frac{1}{\tau}\frac{\propto}{V}\,(P)$ For bimolecular reaction of the form A + B -> C [p] (order 1 reaction)

$(MA) = \frac{1}{\tau}\,(P)$ For monomolecular reaction of the form A -> B [p] (order 0 reaction)

With τ, α and V corresponding to HSIM iteration step (100 µs=10-4 sec.), experimental proportionality factor, and protosensor volume, respectively.

Computed absorbance and fluorescence maps:

In order to predict system's fluorescence and absorbance outputs *in silico* depicted in Figure 3B and 6A, we needed to calibrate computed output concentration to experimental values. This could be achieved by generating experimental calibration curves, which could be then analyzed to yield a mathematical relation between concentration and signal (**Appendix Figure S10**).

3. Microscopic size dispersion measurements

To assess the size dispersion of protosensors, we took random size calibrated microphotograph of protosensor preparations using a Leica DMIL inversed microscope equipped with a 40x lens and a Canon 750D camera mounted on a phototube. Microphotographs were then processed using ImageJ software and a custom script, which allowed for automated size analysis of protosensors. Size dispersion figure were plotted and fitted with using Matlab software

```
setBatchMode(true);
function action(input, output, filename) {
        open(input + filename);
        run("8-bit");
        run("Enhance Contrast", "saturated=4 normalize");
        run("Threshold", "method=Default white");
        run("Watershed");
        run("Set Measurements...", "area mean center perimeter fit shape display redirect=None decimal=5");
        run("Set Scale...", "distance=852.26 known=50 pixel=1 unit=µm global");
        run("Analyze Particles...", "size=.00-1 circularity=0.10-1.00 show=[Overlay Outlines] display exclude clear include");
        saveAs("PNG", output+filename);

        saveAs("Results", output+filename+"results.csv" );
        run("Clear Results");

        close();
}
input = "/tmp/input/";
output = "/tmp/output/";
list = getFileList(input);
for (i = 0; i < list.length; i++)
        action(input, output, list[i]);
setBatchMode(false);
```

.

4. Supplementary references

Afanasyeva, M. S., Taraban, M. B., Purtov, P. A., Leshina, T. V. & Grissom, C. B. Magnetic spin effects in enzymatic reactions: radical oxidation of NADH by horseradish peroxidase. *J. Am. Chem. Soc.* **128,** 8651–8658 (2006).

Aksimentiev, A. & Schulten, K. Imaging α-Hemolysin with Molecular Dynamics: Ionic Conductance, Osmotic Permeability, and the Electrostatic Potential Map. *Biophys. J.* **88,** 3745–3761 (2005).

Arita, N. O., Cohen, M. F., Tokuda, G. & Yamasaki, H. Fluorometric Detection of Nitric Oxide with Diaminofluoresceins (DAFs): Applications and Limitations for Plant NO Research. in *Nitric Oxide in Plant Growth, Development and Stress Physiology* (eds. Lamattina, L. & Polacco, J. C.) **5,** 269–280 (Springer Berlin Heidelberg, 2007).

Dean, J. V. & Harper, J. E. The Conversion of Nitrite to Nitrogen Oxide(s) by the Constitutive NAD(P)H-Nitrate Reductase Enzyme from Soybean. *Plant Physiol.* **88,** 389–395 (1988).

Espey, M. G., Miranda, K. M., Thomas, D. D. & Wink, D. A. Distinction between nitrosating mechanisms within human cells and aqueous solution. *J. Biol. Chem.* **276,** 30085–30091 (2001).

Hansen, N. & Ostermeier, A. Completely derandomized self-adaptation in evolution strategies. *Evol. Comput.* **9,** 159–195 (2001).

Kojima, H. *et al.* Detection and imaging of nitric oxide with novel fluorescent indicators: diaminofluoresceins. *Anal. Chem.* **70,** 2446–2453 (1998).

Liu, X. *et al.* Application of carbon fiber composite minielectrodes for measurement of kinetic constants of nitric oxide decay in solution. *Nitric Oxide* **23,** 311–318 (2010).

Li, H., Kundu, T. K. & Zweier, J. L. Characterization of the Magnitude and Mechanism of Aldehyde Oxidase-mediated Nitric Oxide Production from Nitrite. *J. Biol. Chem.* **284,** 33850–33858 (2009).

Nagle JF & Tristram-Nagle S. Structure of lipid bilayers. *Biochim Biophys Acta.* 159–95. (2000).

Olasehinde & et al. Reaction Kinetics for Nitrosation of DAF-2 in Air Saturated Nitric Oxide Solution. *Nature & Science* p129 (2012)

Planchet, E. & Kaiser, W. M. Nitric oxide (NO) detection by DAF fluorescence and chemiluminescence: a comparison using abiotic and biotic NO sources. *J. Exp. Bot.* **57,** 3043–3055 (2006).

Rizk, A., Batt, G., Fages, F. & Soliman, S. A general computational method for robustness analysis with applications to synthetic gene networks. *Bioinformatics* **25,** i169–i178 (2009).

Singh, R. *et al.* Catalase-peroxidases (KatG) exhibit NADH oxidase activity. *J. Biol. Chem.* **279,** 43098–43106 (2004).

Stillwell, W. *An introduction to biological membranes: from bilayers to rafts*. (Elsevier/Academic Press, 2013).
Teh, S.-Y., Khnouf, R., Fan, H. & Lee, A. P. Stable, biocompatible lipid vesicle generation by solvent extraction-based droplet microfluidics. *Biomicrofluidics* **5,** 044113 (2011).

Watanabe, R. *et al.* Arrayed lipid bilayer chambers allow single-molecule analysis of membrane transporter activity. *Nat. Commun.* **5,** (2014).

Yokota, K. & Yamazaki, I. Analysis and computer simulation of aerobic oxidation of reduced nicotinamide adenine dinucleotide catalyzed by horseradish peroxidase. *Biochemistry (Mosc.)* **16,** 1913–1920 (1977).

Yorita, K. *et al.* Conversion of L-lactate oxidase to a long chain alpha-hydroxyacid oxidase by site-directed mutagenesis of alanine 95 to glycine. *J. Biol. Chem.* **271,** 28300–28305 (1996).

II. Appendix Figures and Tables.

Molecule designation	Stock solution	Concentration of use (batch/protosensors)		
		GluONe	LacOH	GluNOx
NAD+	50 mM PBS	250 µM / 4 mM	250 µM/ 5 mM	100 µM /2 mM
NADH	50 mM PBS	-	-	-
Acetone	100 mM PBS	1 mM (unless specified)	-	-
Ethanol	100 mM PBS	-	20 mM (unless specified)	-
Glucose	50 mM PBS	1 mM (unless specified)	-	5 mM (unless specified)
NO_3	50 mM PBS	-	-	5 mM (unless specified)
Lactate	100 mM PBS	-	0.5 mM (unless specified)	-
Isopropyl alcohol	100 mM PBS	-	-	-
G1DH	3.4 U/µl PBS	8.5 U/ml=0.354 µM / 7.4 µM	-	138 U/ml=5.7546 µM / 127 µM
ADH	55.7 U/ml PBS	0.2785 U/ml=14.06 µM / 221 µM	0.2785 U/ml=14.06 µM / 317 µM	-
AO	0.1 U/µl PBS	0.75 U/ml=0.02725 µM / 0.59 µM	-	-
NR	1U/ml PBS	-	-	4.2 µM= 0.5 U/ml / 92 µM
HRP	10 U/ml PBS	0.015 U/ml=0.00104 µM / 0.0208 µM	0.05 U/ml=0.00347 µM / 0.0754 µM	-
LO	6.425 U/µl PBS	-	0.1 U/ml=1.12 µM / 23.4 µM	-
Hemolysin	250 µM PBS	1 µM	1 µM	1 µM
Resazurin	10 mM water	50 µM / 1 mM	-	-
ABTS	10 mM PBS	-	100 µM / 2 mM	-
DAF-2	5 mM DMSO	-	-	10 µM / 200 µM

Appendix Table S1: Stock solutions and concentrations used in this study. All chemicals and enzymes were purchased from Sigma Aldrich. G1DH: Glucose-1-dehydrogenase, ADH: Alcohol dehydrogenase, AO: Alcohol oxidase, NR: Nitrate/Nitrite reductase, HRP: Horseradish peroxidase, LO: Lactate oxidase, ABTS: 2,2'-azino-bis(3-ethylbenzothiazoline-6-sulphonic acid), DAF-2: 4,5-Diaminofluorescein. See Table 2 below for more information about enzymes used in this study. Stock solutions were kept at -30°C until use. Resorufin and ABTS solution were prepared the same day of the assays or kept no longer than a week.

Enzyme/reaction	Organisms	Substrat 1	Km (mM)	Substrat 2	Km (mM)	Kcat1 (/s)	Kcat2 (/s)	Source	Mechanism
Nitrate Reductase (nitrate-> nitrite) EC 1.7.1.1	*Arabidopsis thaliana*	NADH	0.004	NO_3^-	0.015	-	210	BRENDA	Random bi-bi
Nitrate Reductase (nitrite-> NO) EC 1.7.1.1	*Arabidopsis thaliana*	NADH	0.004	NO_2^-	0.0074	-	2	Plantchet & Kaiser, 2006<br>Dean & Harper, 1988	
Glucose 1-Dehydrogenase EC 1.1.1.47	*Pseudomonas sp.*	NAD+	80	Glucose	0.86	200	400	BRENDA	Ordered bi-bi
Alcohol Dehydrogenase EC 1.1.1.1	*Equs Caballus*	NADH	0.0025	Acetone / acetaldehyde	135 / 6	0.717	0.33 / 31.8	BRENDA	Ordered bi-bi
Alcohol Dehydrogenase EC 1.1.1.1 reverse	*Equs Caballus*	NAD+	0.34	Isopropanol	268	0.41	0.75		
Alcohol Oxydase EC 1.1.3.13	*Candida sp.*	Isopropanol	10	-	-	150	-	BRENDA	Ping-pong
Horseradish peroxidase 1.11.1.7	*Armoracia rusticana*	amplex Red	0.081	H_2O_2	0.005	240	-	BRENDA	Ping-pong
		ABTS	0.18	H_2O_2	0.005	760	-		
		NADH	0.012	H_2O_2	0.005	0.009	-	Yokota & Yamazaki, 1977<br>Singh et al., 2004<br>Afanasyeva et al., 2006	
Lactate Oxidase 1.13.12.4	*Pediococcus sp.*	(S)-Lactate	0.2	-	-	-	283.3	Yorita et al., 1996<br>BRENDA	Ping-pong
Alcohol Dehydrogenase EC 1.1.1.1	*Equs Caballus*	NAD	0.0074-0.01	Ethanol	2.46	308	-	BRENDA	Ordered bi-bi
NO decay	*N.A.*	NO	-	-	-	1.9.10-3	-	Liu et al., 2010	N.A.
NO reaction	*N.A.*	O_2	0.001	-	-	2	-	Li et al., 2009<br>Dean & Harper, 1988<br>Kojima et al., 1998<br>Espey et al., 2001<br>Plantchet & Kaiser, 2006<br>Arita et al., 2007<br>Olasehinde et al., 2012	N.A.
		NO_2	0.001	-	-	2000	-		
		DAF-2	0.001	-	-	6.28	-		
N_2O_3 reaction	*N.A.*	DAF-2	0.001	-	-	2000	-		

Appendix Table S2: Enzymatic and kinetic parameters of biochemical species used in this study

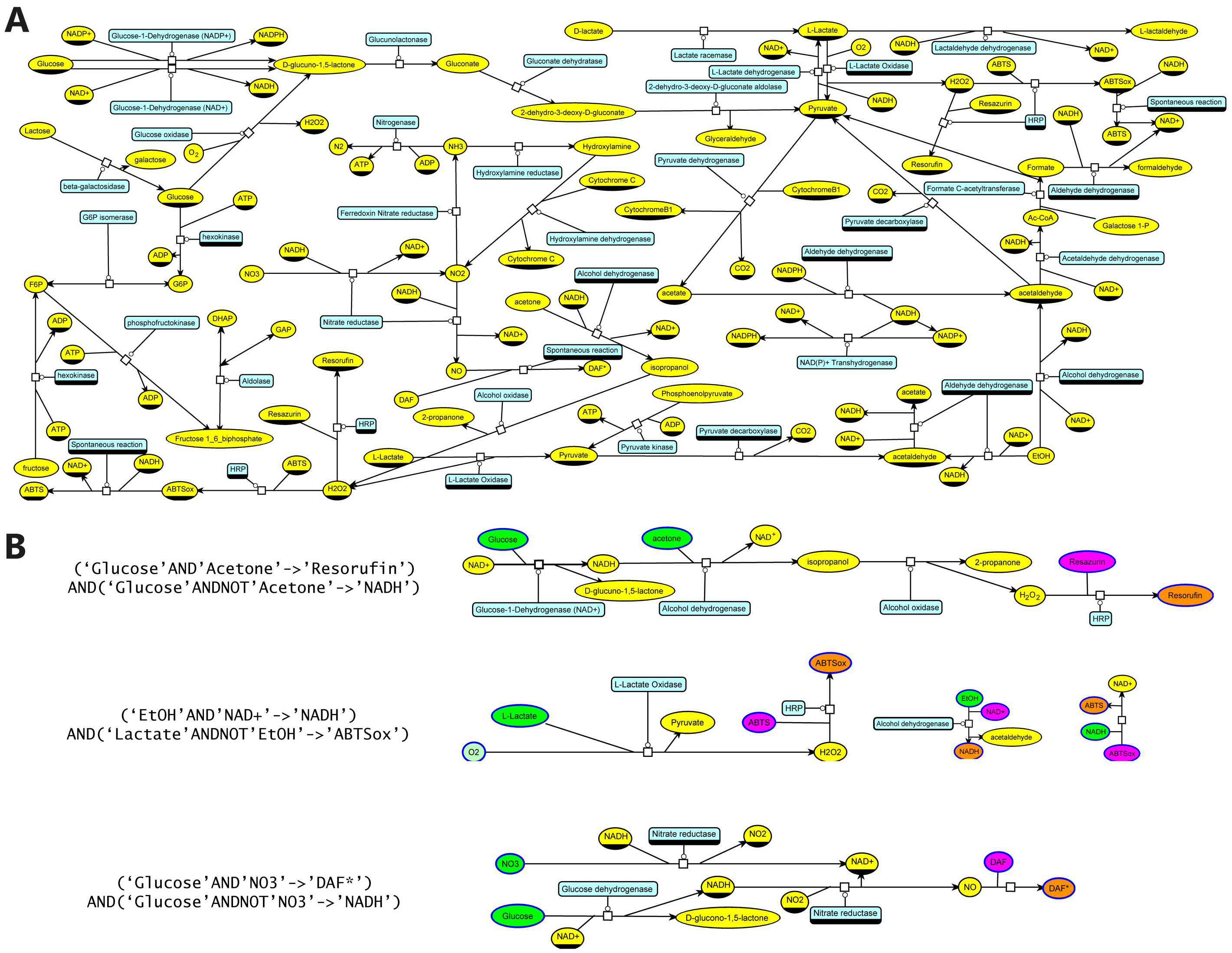

Appendix Figure S1: Automated biochemical implementation of user defined enzymatic Boolean logic gates from natural metabolic networks. To generate the synthetic biochemical circuits described in this study, we performed an organism agnostic search of all the sets of natural biochemical networks with overlapping enzymes, substrates or products related to the inputs and outputs of the different biochemical circuit we aimed at designing. The BRENDA database was queried for biochemical network containing enzymes, substrate or product related to glucose, acetone, NADH, resorufin, lactate, ethanol, and NOx metabolism (*i.e.* acetate fermentation, glycolysis, propanol degradation, disaccharide metabolism, ethanol fermentation, lactate fermentation, L-lactaldehyde degradation, methane metabolism, non-pathway related, NAD metabolism, Entner Doudoroff pathway, Nitrate assimilation, as well as non-pathway related peroxidase catalyzed reactions) and SBML files of these networks were downloaded from the BRENDA web interface (https://www.brenda-enzymes.org/search_result.php?a=200). We then used Silicell Maker to combine them into one large SBML network via the ‘share clones’ command (Computer Code EV1). This large network (**A**) was then used as input and mined using the program NetGate to identify all biochemical logic gates of =<2 reactions (‘Formula -> Extract Gates’ command). In this first step, all the possible implementations of logic gates present in the input network are enumerated, and 775 logic gates were identified in this case. An implementation of a logic gate is a subnetwork where appropriate biomolecular inputs and output are identified. The program NetBuild was then used (‘Formula -> Enter Formula...’ command) to find specific biochemical implementation corresponding to user-defined Boolean logic specifications. In this second step, all enumerated implementations are checked against the given truth tables and the gates found are sorted. The algorithm found unique implementations satisfying GluONe, LacOH and GluNOx biochemical logic from the input network (**B**). Increasing the number of reactions/gates would augment the solution space by yielding more implementations of higher complexity but would be considerably more computationally expensive. To increase robustness and ease experimental and computational work for the purpose of this study, we pursued the smallest reactions set capable of recapitulating a specific logic formula.

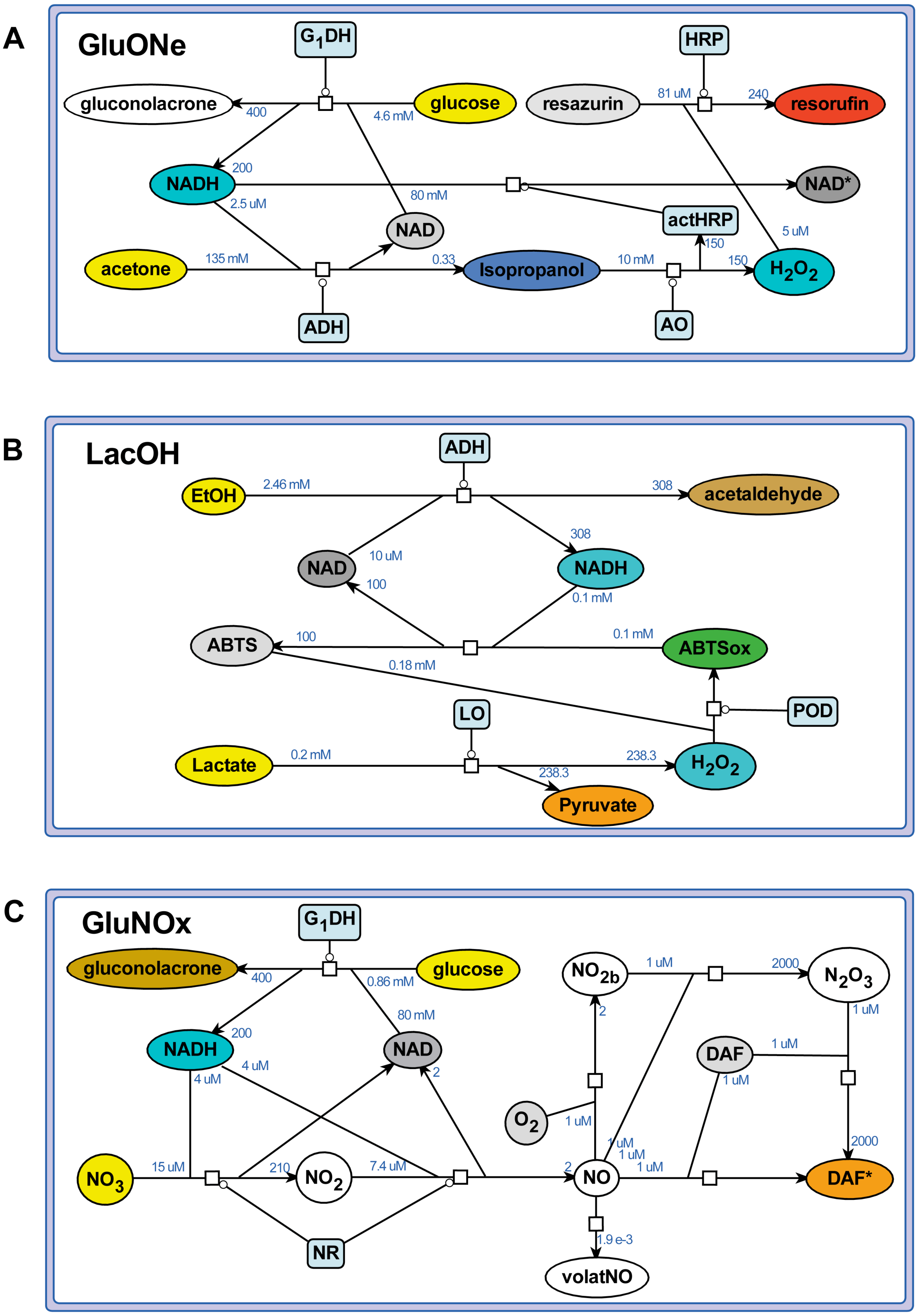

Appendix Figure S2: Topology of biochemical circuits with kinetic parameters that were designed and used in this study. Metabolites corresponding to systems inputs are depicted in yellow. (**A**) The GluONe system takes Glucose and Acetone as inputs in the medium and applies AND and N-Imply Boolean logic to inputs, to generate absorbance and fluorescent output signal in the molecular form of NADH and Resorufin, respectively. It comprises 4 different enzymes and 2 different metabolites. (**B**) The LacOH system takes Lactate and Ethanol as inputs in the medium and applies N-Imply Boolean logic to inputs, to generate an absorbance and colorimetric output signal in the molecular form of oxidized ABTS. It comprises 3 different enzymes and 2 different metabolites. (**C**) The GluNOx system takes Glucose and NOx as inputs in the medium and applies AND Boolean logic to inputs, to generate a fluorescent output signal in the molecular form of nitrosylated DAF-2. It comprises 3 different enzymes and 2 different metabolites. SBML files corresponding to the circuits can be found attached as Supplementary Materials files.

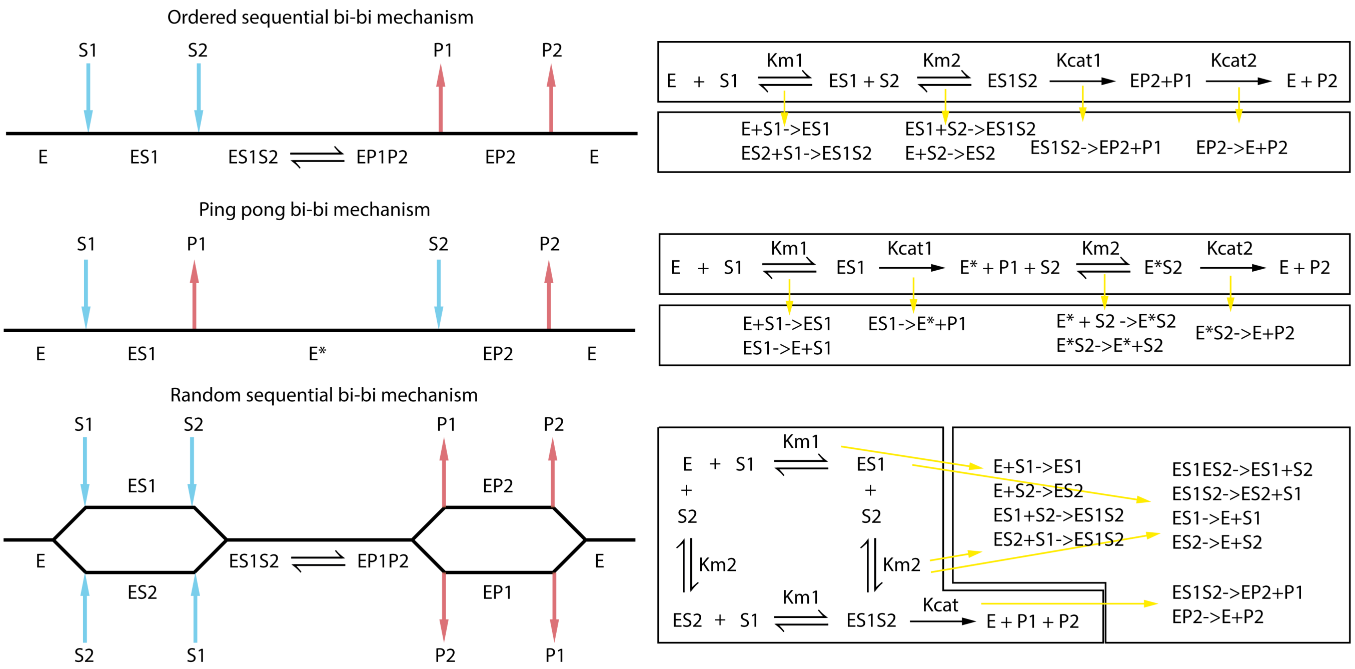

Appendix Figure S3: Multisubstrate enzymatic mechanisms describing biochemical reactions and corresponding HSIM models equations.

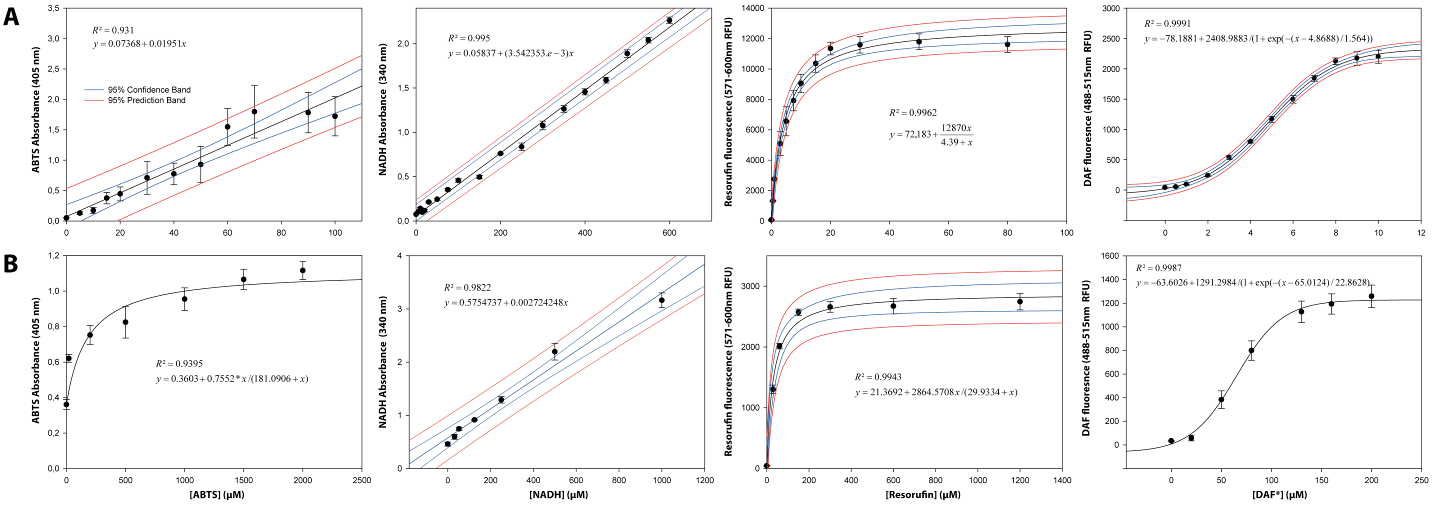

Appendix Figure S4: Experimental calibration curves used to compute output signals. In order to obtain a mathematical relation between concentration of outputs and experimental fluorescence and absorbance measurements, we measured signals from samples spiked with known concentration of output molecules. (**A**) Output molecular signal in PBS buffer (**B**) Output molecular signals in protosensors. Depicted are the mathematical formulas used for calculations.

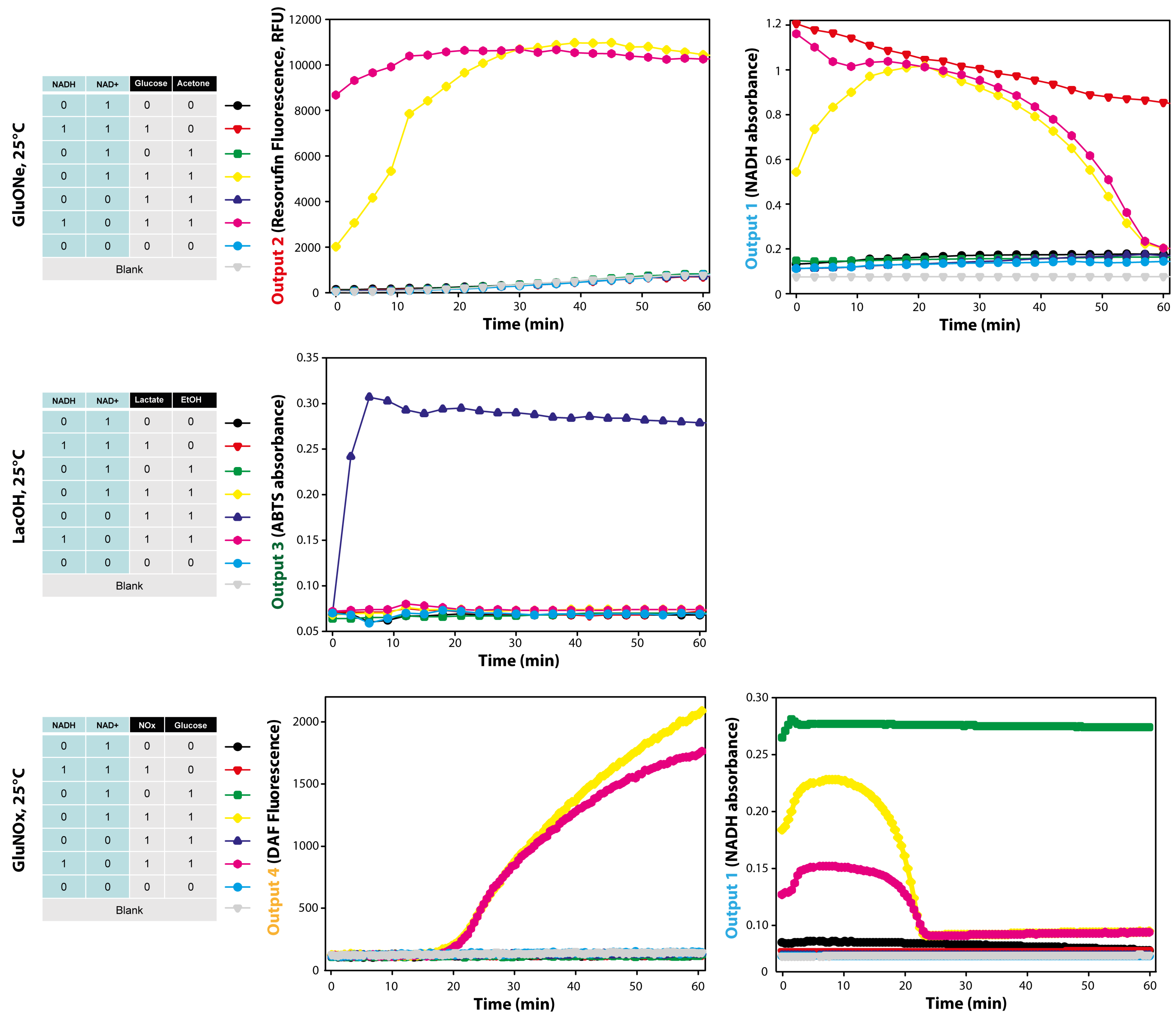

NADH	NAD+	Glucose	Acetone
0	1	0	0
1	1	1	0
0	1	0	1
0	1	1	1
0	0	1	1
1	0	1	1
0	0	0	0
Blank			

NADH	NAD+	Lactate	EtOH
0	1	0	0
1	1	1	0
0	1	0	1
0	1	1	1
0	0	1	1
1	0	1	1
0	0	0	0
Blank			

NADH	NAD+	NOx	Glucose
0	1	0	0
1	1	1	0
0	1	0	1
0	1	1	1
0	0	1	1
1	0	1	1
0	0	0	0
Blank			

Appendix Figure S5: Detailed experimental kinetic characterization of synthetic biochemical circuits *in vitro*. Enzymes and metabolites were mixed in p96 100µl wells in PBS, homogeneized via smooth agitation, and inputs were added last. Kinetic measurments were performed on a Synergy H1 plate reader, under slow agitation at 25°C.

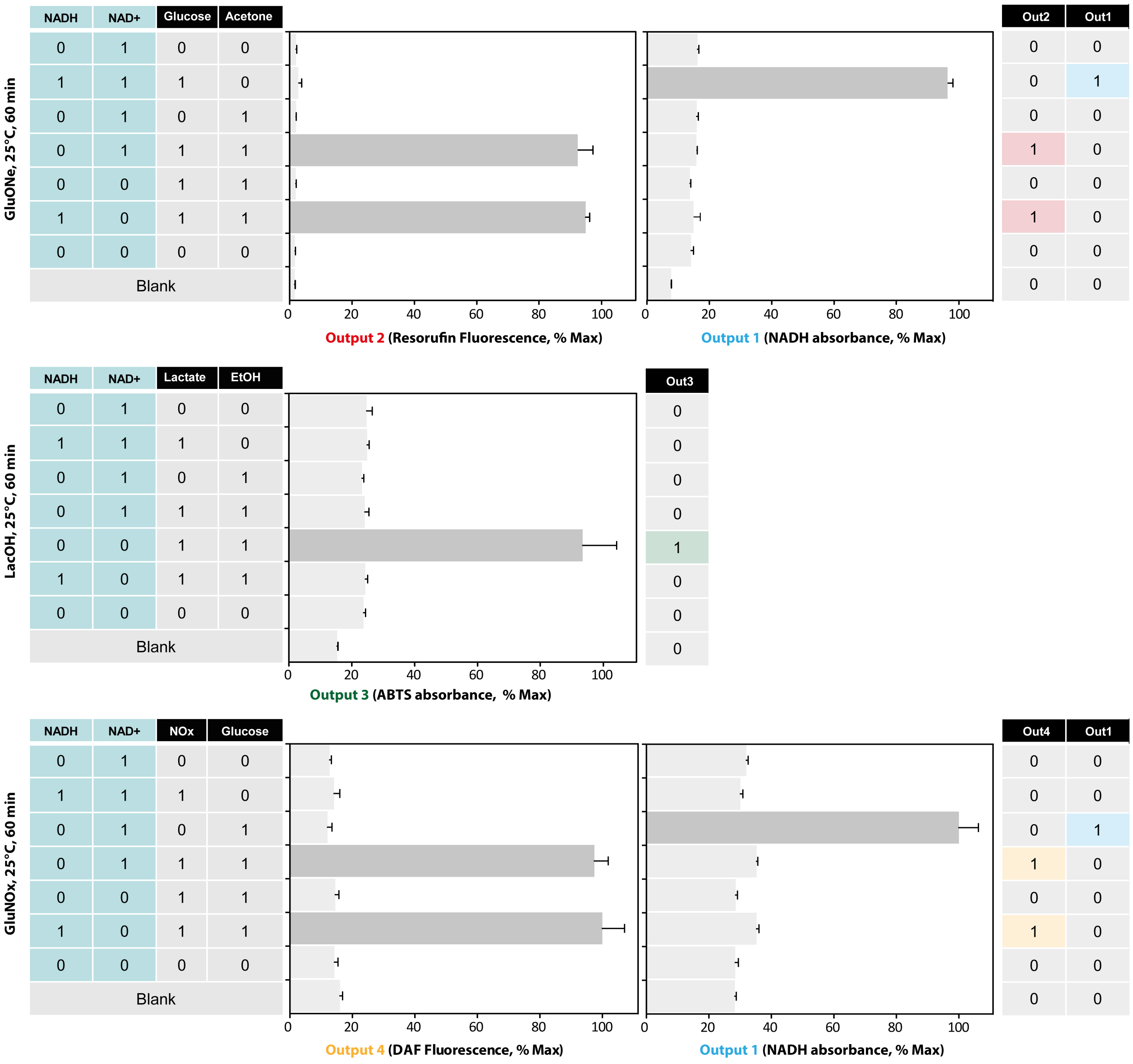

Appendix Figure S6 : Detailed experimental logic characterization of synthetic biochemical circuits *in vitro.* In this figure are depicted measurements obtained as previsouly detailed.

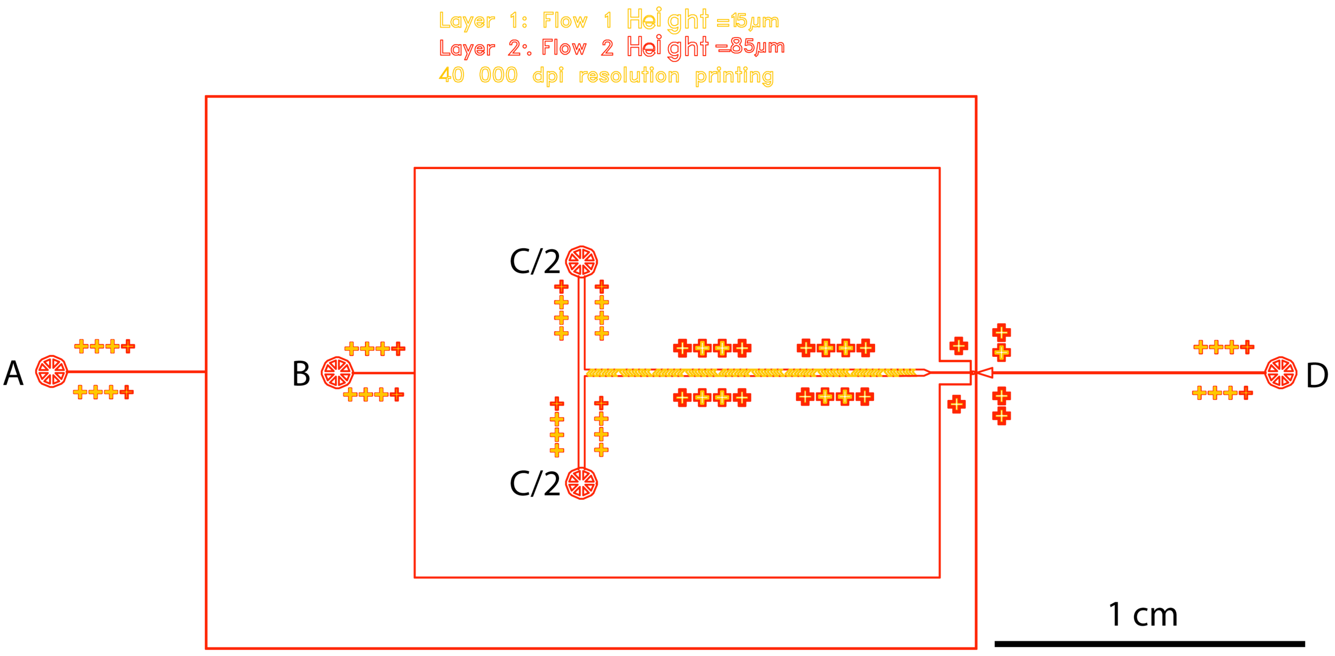

Appendix Figure S7: Microfluidic chip used in this study to generate protosensors. (A) Buffer (10% v/v methanol, 15% w/v glycerol, 3% w/v pluronic F68 in PBS), 1 µl/min (B) DPPC dissolved in oleic acid, 0.4 µl/min (C) Enzymes in PBS, 0.4 µl/min (D) Out.

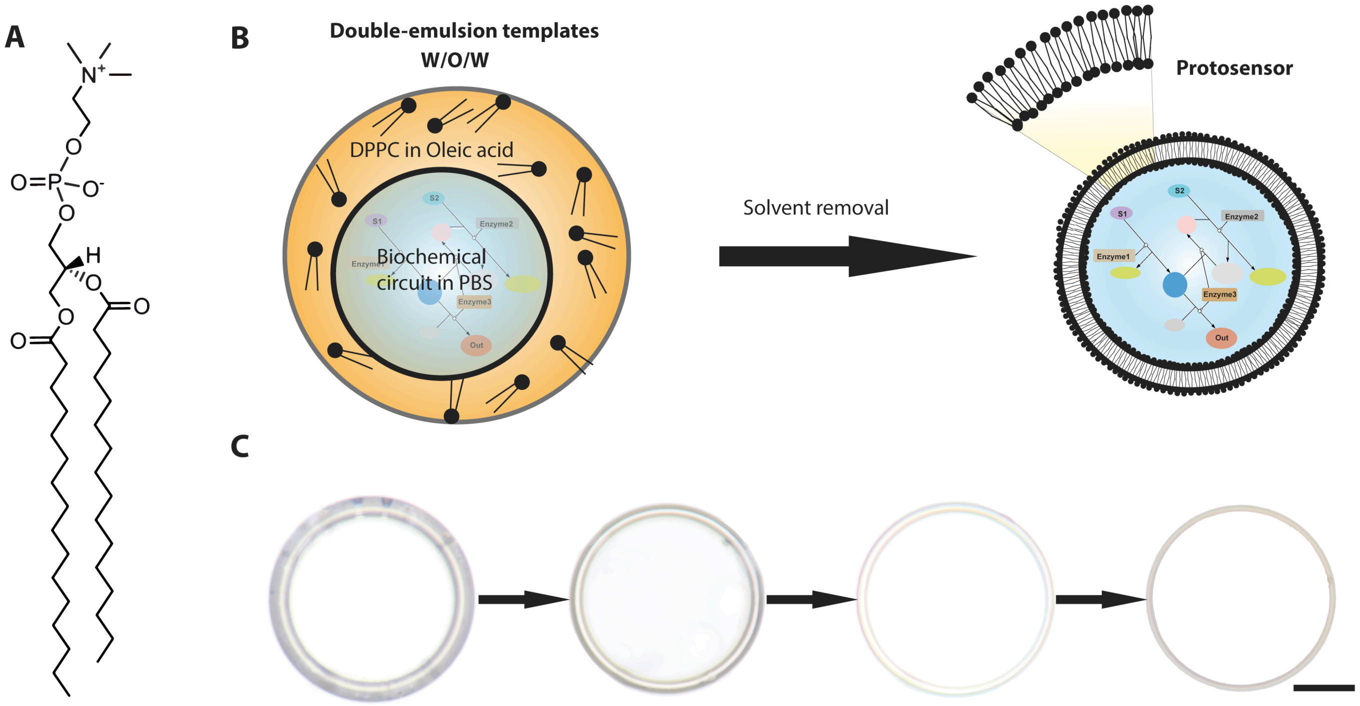

Appendix Figure S8: Double emulsion template for protosensor directed self-assembly. (**A**) The microfluidic flow-focusing droplet generation device generates double emulsion templates, and (**B**) Oleic acid is then extracted to generate protosensors. (**C**) Visualization of the extraction process at 0, 1, 2 and 3 hours (Scale bar=2.5µm).

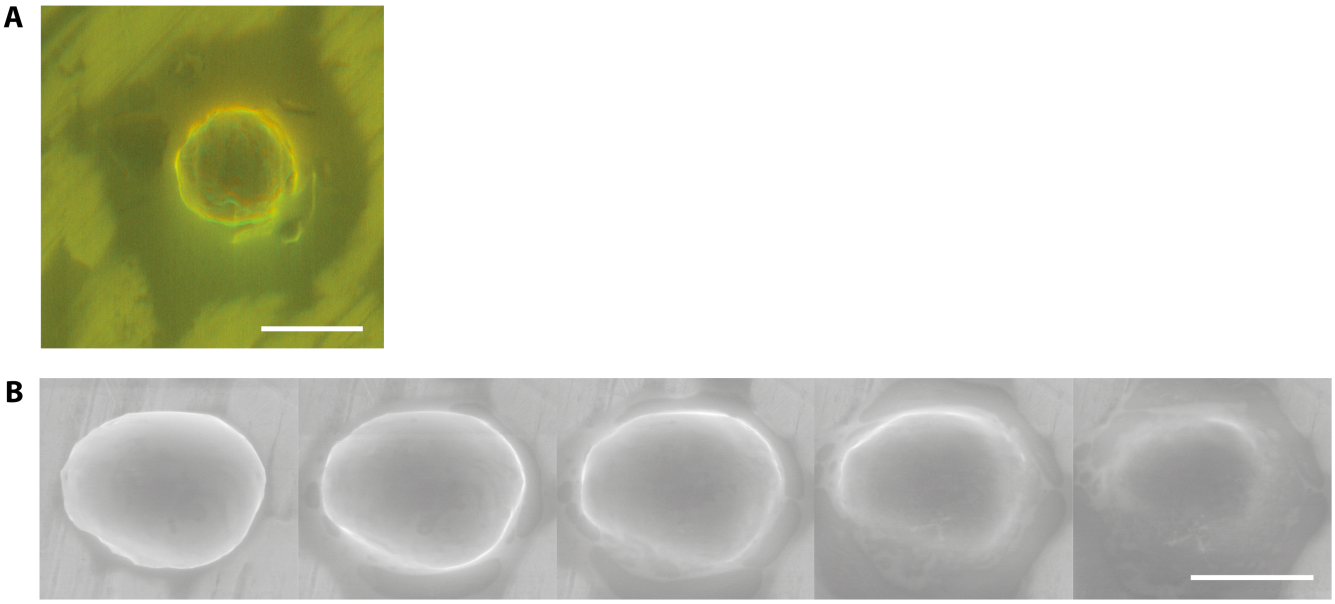

Appendix Figure S9: Environmental Scanning Electron Microscopy (ESEM) photomicrograph of protosensors. DPPC vesicles were fixed overnight at 4°C in 2.5% glutaraldehyde solution in PBS, and then washed with water prior to direct observation. (**A**) Stereoscopic micrograph of individual protosensor (**B**) Kinetic visualization of electron beam interacting with a protosensor. From left to right: 10 seconds were sufficient to melt the DPPC bilayer and release intra-vesicular content. (Scale bar=10µm)

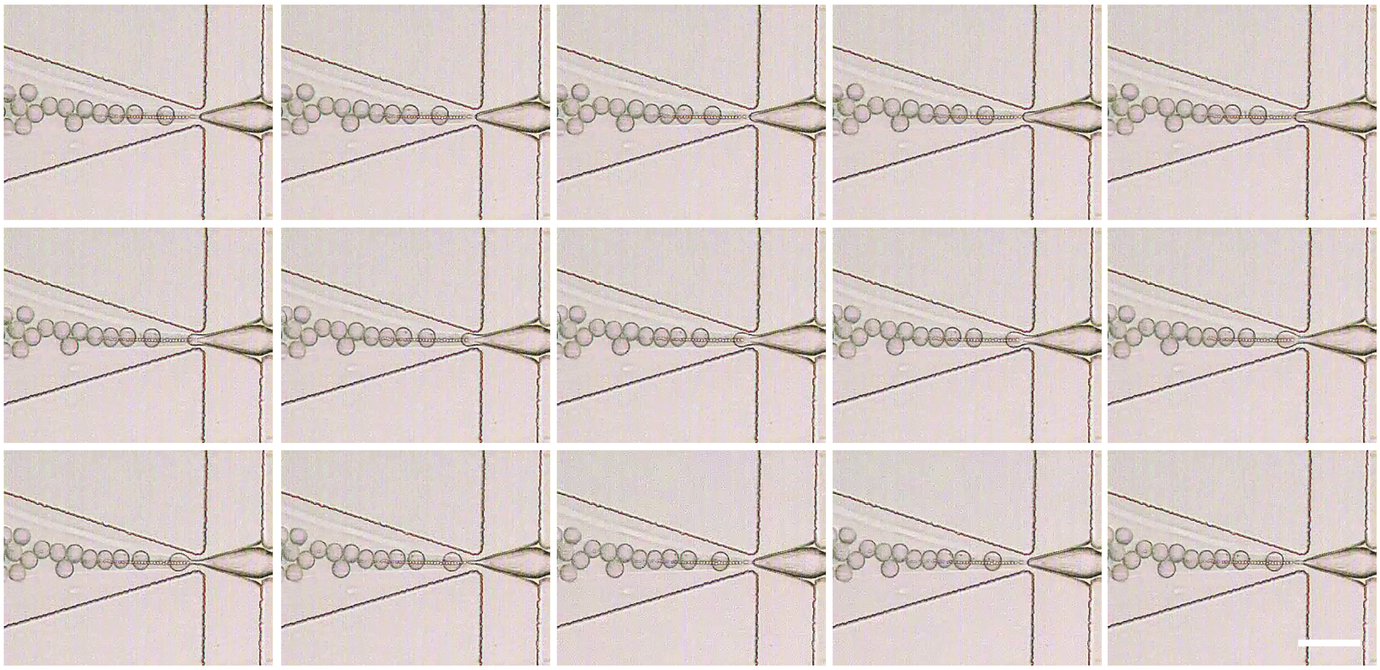

Appendix Figure S10: Time-lapse photomicrograph of protosensors fabrication within microchannels. (Read from left to right, top to bottom). The movie (**Movie EV1**) was recorded at 20 000 FPS and the time-lapse corresponds to ~500 µs. (Scale bar=40 µm)

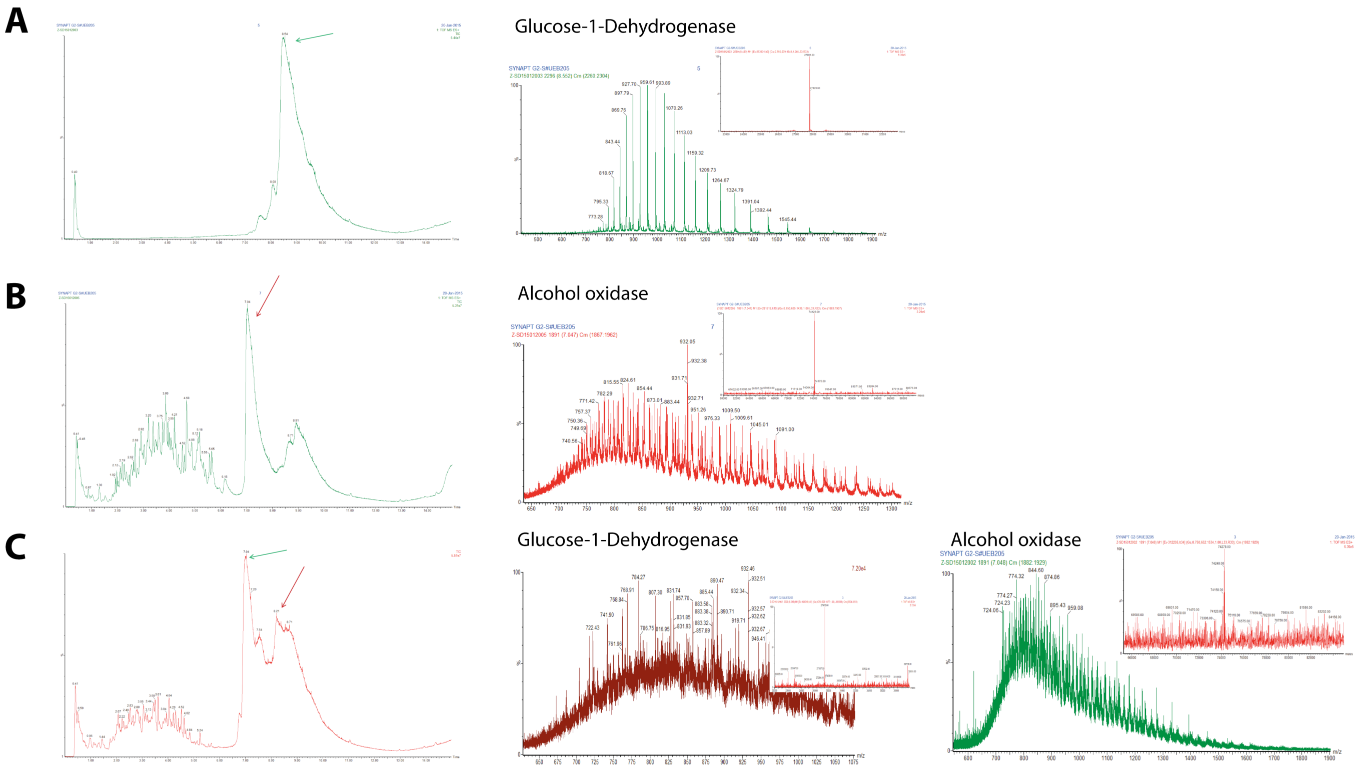

Appendix Figure S11: UPLC-Mass spectrometry experiments to assay enzyme encapsulation in protosensors. Experiments were performed on an Acquity UPLC (Waters) coupled with a TSQ Quantum (Thermofischer). Briefly, we used a Kinetex C18 column (100x2.1mm 2.6um) with H2O+0.01% formic acid and acetonitrile+0.01% formic acid as eluents and a flow rate of 0.5ml/ml, with ESI+ detection. (**A**) Chromatogram of G1DH enzyme in PBS buffer and MS spectra of main peak, which enables to identify the enzyme with a mass corresponding to literature (~30kDa) (**B**) Chromatogram of AO enzyme in PBS buffer and MS spectra of main peak, which enables to identify the enzyme with a mass corresponding to literature (~74kDa) (**C**) Chromatogram of protosensor encapsulating G1DH and AO enzymes in PBS buffer, and MS spectra of main peaks, which enables to identify the two enzymes.

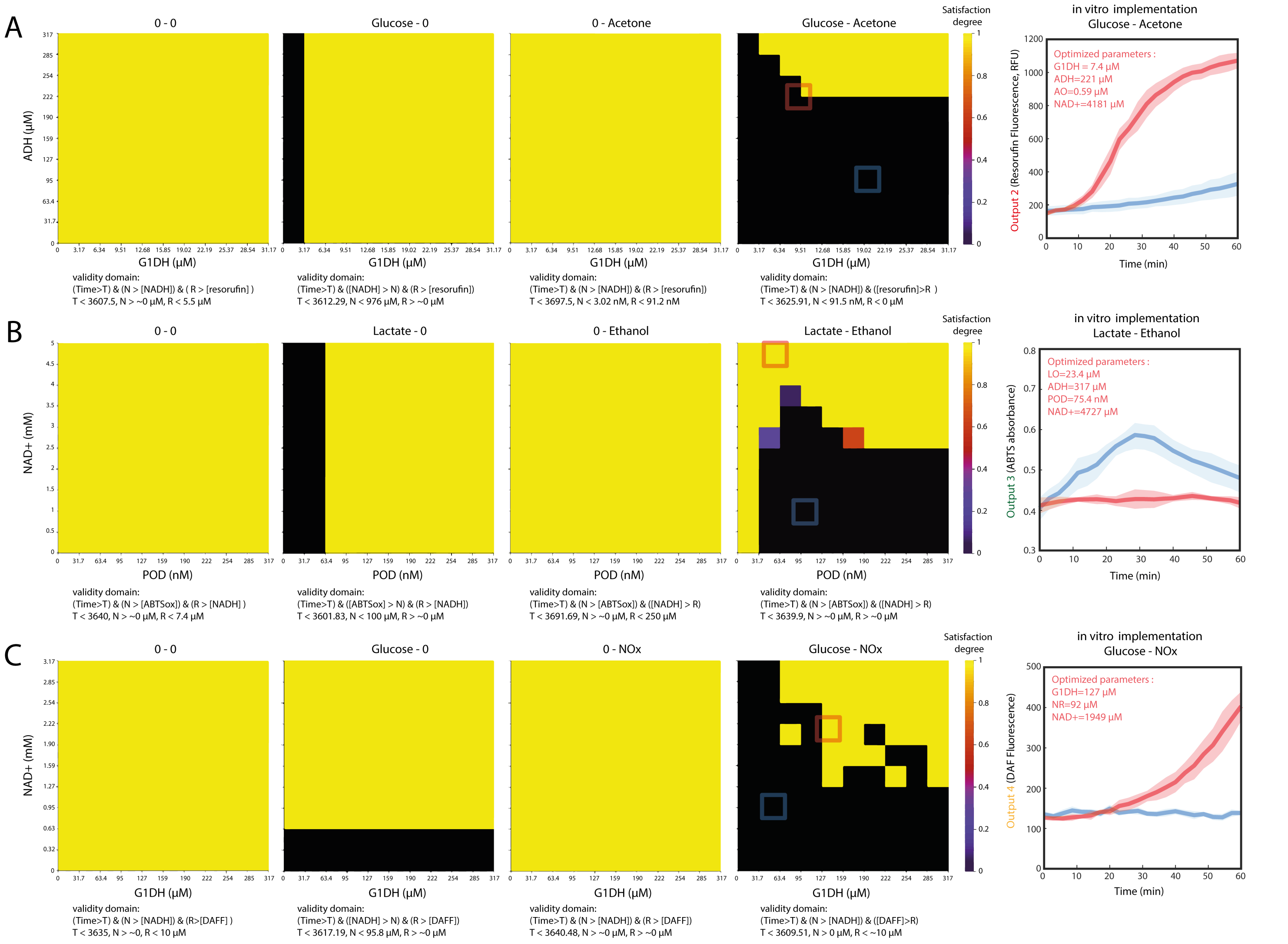

Appendix Figure S12: Mapping satisfaction degree landscape for (A) GluOne, (B) LacOH and (C) GluNOx biochemical circuits in protosensors. The satisfaction degrees of the temporal logic formulas specifying the behaviour of the protosensors over 5 minutes were computed while varying the two most sensitive parameters of each model (e.g. the initial concentrations of ADH and G1DH for GluONe) for each combination of inputs. The temporal logic constraints about the concentration thresholds of the outputs at steady state are depicted below each maps along with the validity domains of their variables. The initial concentration parameters were then optimized using the CMAES evolutionary algorithm implemented in BIOCHAM. We verified experimentally the kinetic response of protosensors implemented with specific concentration of enzymes corresponding to the colored squares on the map (Right).

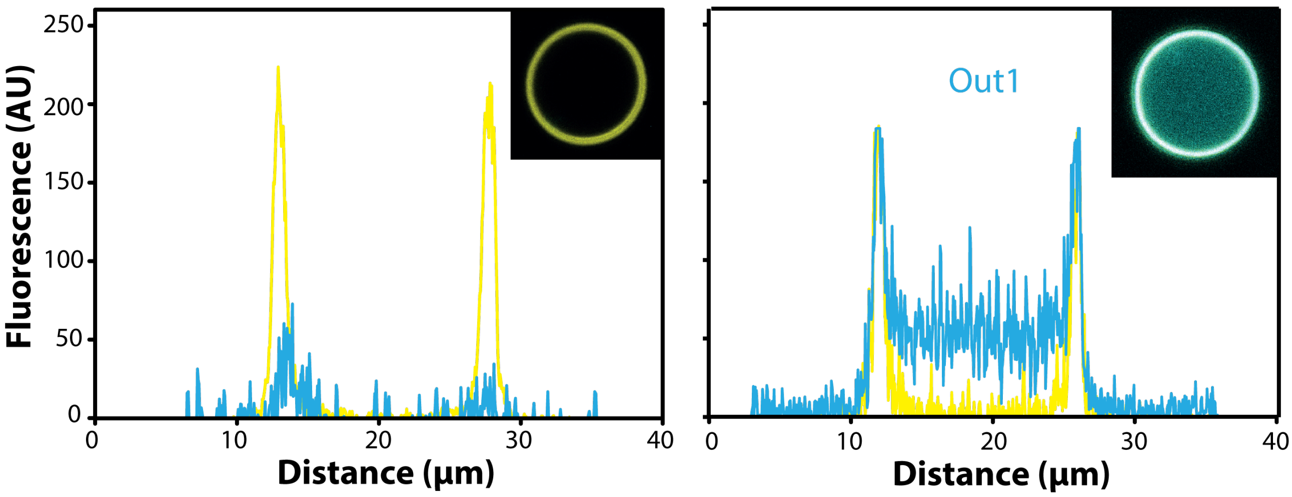

Appendix Figure S13: Experimental validation of GluONe Out1 using confocal microscopy. We validated "ON" output signals of GluONe protosensors responses at 60 minutes without (left) and after induction with glucose biomarker at the concentration of $1.10^{-4.5}$ M (right). The phospholipid bilayer is stained in yellow using the dye DiC_{18}. We measured the fluorescence of the output signal NADH here shown in blue, using excitation from a 355nm UV laser.

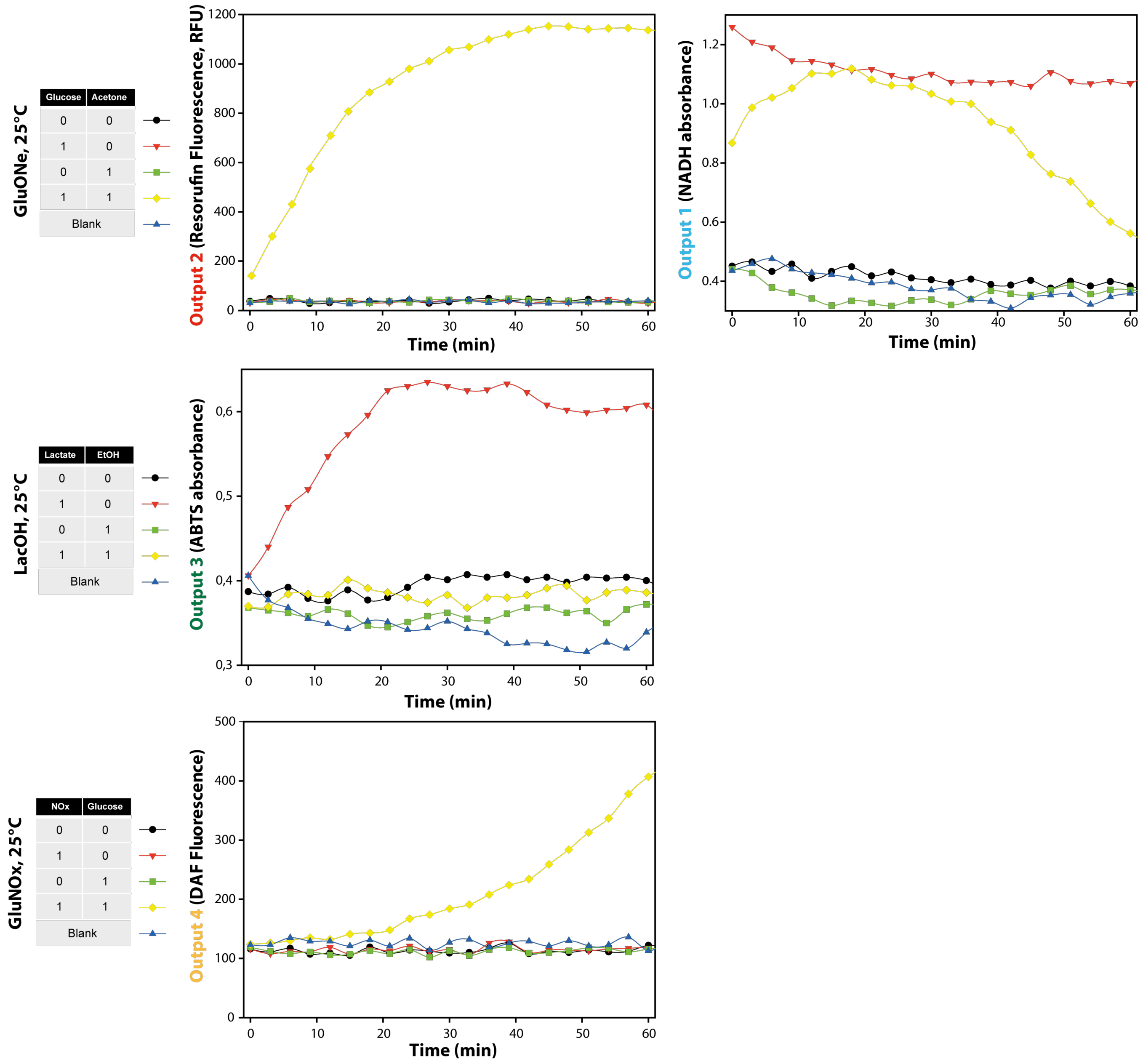

Appendix Figure S14: Detailed experimental kinetic characterization of synthetic biochemical circuits in protosensors operating in urine.

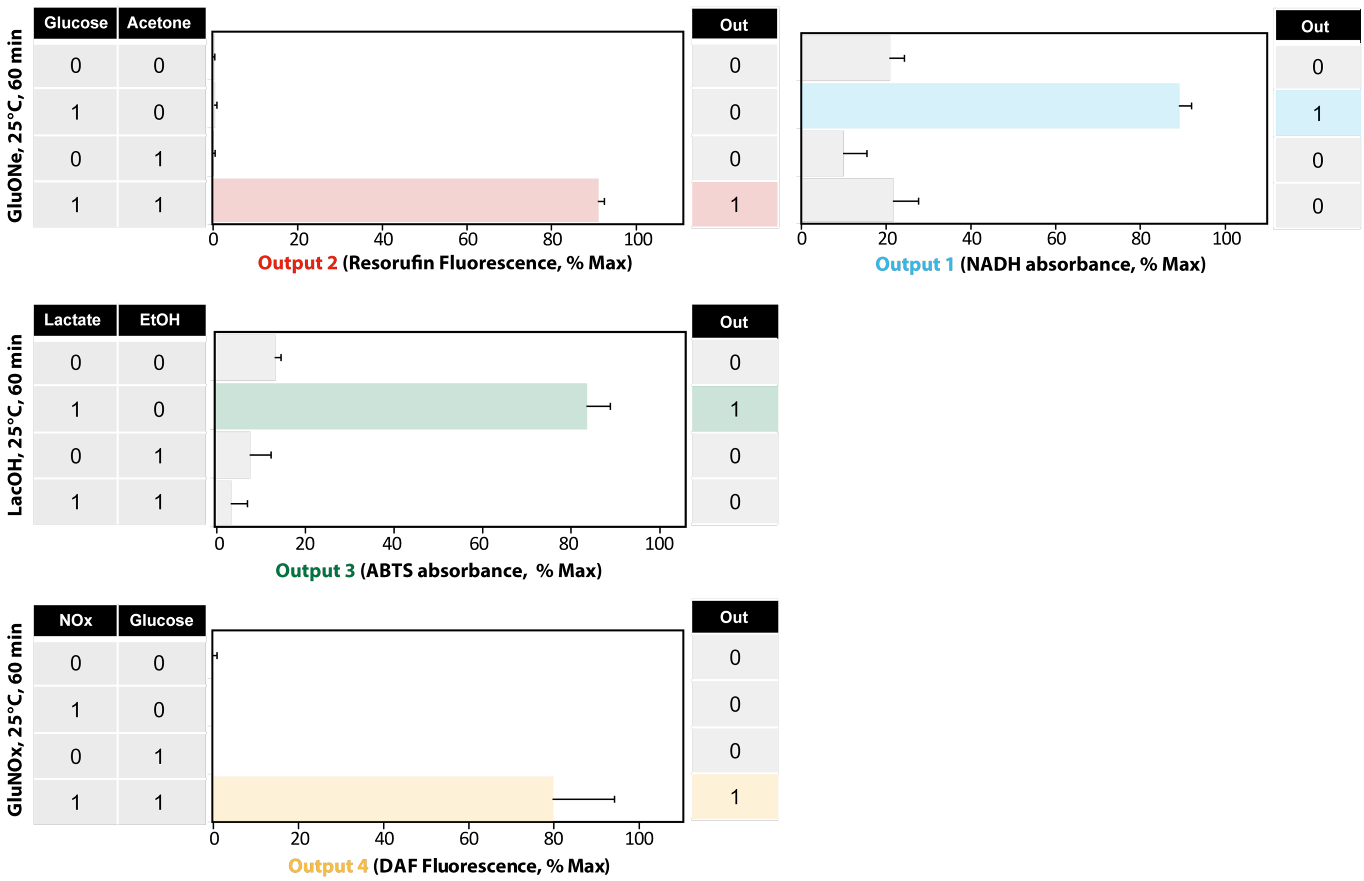

Appendix Figure S15: Experimental truth tables of protosensors operating in PBS.

III. HSIM code for models used in this study

1. GluONe batch mode

```
title = "GluONe_Batch";
geometry = 1000:1000;

metabolite
        'glucose',
        'gluconolacrone',
        'G_1DH',
        'acetone',
        'isopropanol',
        'ADH',
        'NADH',
        'NAD',
        'resazurin',
        'resorufin',
        'HRP',
        'H_2O_2',
        'AO',
        'NADN',
        'HRP2';

display (
        'NADN');

init (1 mM, 'glucose');
init (1 mM, 'acetone');

//      R 5:

metabolite Cfa5 hide, Cia5 hide, Cib5 hide;
'HRP' + 'H_2O_2' -> Cia5 [0.814815];
Cia5 -> 'HRP' + 'H_2O_2' [0.0024];
Cia5 + 'resazurin' -> Cib5 [0.55];
Cib5 -> Cia5 + 'resazurin' [0.0001];
Cib5 -> 'HRP' + 'resorufin' [0.024];

//      R 4:

metabolite Cf4 hide;
'HRP2' + 'NADH' -> Cf4 [0.00020625];
Cf4 -> 'HRP2' + 'NADH' [9e-008];
Cf4 -> 'HRP2' + 'NADN' [9e-007];

//      R 3:

metabolite Cf3 hide;
'AO' + 'isopropanol' -> Cf3 [0.004125];
Cf3 -> 'AO' + 'isopropanol' [0.0015];
metabolite Cio3 hide;
Cf3 -> Cio3 + 'H_2O_2' [0.015];
Cio3 -> 'AO' + 'HRP2' [1];

//      R 2:

metabolite Cfa2 hide, Cia2 hide, Cib2 hide;
'ADH' + 'NADH' -> Cia2 [6.72222e-007];
Cia2 -> 'ADH' + 'NADH' [3.3e-006];
Cia2 + 'acetone' -> Cib2 [0.077];
Cib2 -> Cia2 + 'acetone' [7e-006];
Cib2 -> Cfa2 + 'NAD' [7e-005];
Cfa2 -> 'ADH' + 'isopropanol' [3.3e-005];

//      R 1:

metabolite Cfa1 hide, Cia1 hide, Cib1 hide;
'G_1DH' + 'NAD' -> Cia1 [0.127907];
```

```
Cia1 -> 'G_1DH' + 'NAD' [0.004];
Cia1 + 'glucose' -> Cib1 [0.0006875];
Cib1 -> Cia1 + 'glucose' [0.002];
Cib1 -> Cfa1 + 'NADH' [0.02];
Cfa1 -> 'G_1DH' + 'gluconolacrone' [0.04];

init (0.354uM, 'G_1DH');
init (14.6uM, 'ADH');
init (250uM, 'NAD');
init (50 uM, 'resazurin');
init (0.001041uM, 'HRP');
init (0.02775uM, 'AO');
init (0.001041uM, 'HRP2');
```

2. GluONe protosensor mode

```
title = "GluONe_protosensor";
geometry = 1500:1500;

metabolite
	'glucose',
	'gluconolacrone',
	'G_1DH',
	'acetone',
	'isopropanol',
	'ADH',
	'NADH',
	'NAD',
	'resazurin',
	'resorufin',
	'HRP',
	'H_2O_2',
	'AO',
	'NADN',
	'HRP2';

compartment {
	geometry = 1000:1000+0+0+0;		// 10000 nm de long, 10000 nm de diametre
	init (7.08uM, 'G_1DH');
	init (292uM, 'ADH');
	init (5000uM, 'NAD');
	init (1000 uM, 'resazurin');
	init (0.02082uM, 'HRP');
	init (0.555uM, 'AO');
	init (0.02082uM, 'HRP2');
}

display (
	'NADN');

init (1 mM, 'glucose');
init (1 mM, 'acetone');

diffusion (glucose) = 5e-3; // Arrayed lipid bilayer chambers allow single-molecule analysis of membrane transporter activity
diffusion (acetone) = 1e-2; // Permeable ~ethanol/H2O, Molecular Biology of the Cell. 4th edition.

//	R 5:

metabolite Cfa5 hide, Cia5 hide, Cib5 hide;
'HRP' + 'H_2O_2' -> Cia5 [0.814815];
Cia5 -> 'HRP' + 'H_2O_2' [0.0024];
Cia5 + 'resazurin' -> Cib5 [0.55];
Cib5 -> Cia5 + 'resazurin' [0.0001];
Cib5 -> 'HRP' + 'resorufin' [0.024];

//	R 4:

metabolite Cf4 hide;
```

```
'HRP2' + 'NADH' -> Cf4 [0.00020625];
Cf4 -> 'HRP2' + 'NADH' [9e-008];
Cf4 -> 'HRP2' + 'NADN' [9e-007];

//		R 3:

metabolite Cf3 hide;
'AO' + 'isopropanol' -> Cf3 [0.004125];
Cf3 -> 'AO' + 'isopropanol' [0.0015];
metabolite Cio3 hide;
Cf3 -> Cio3 + 'H_2O_2' [0.015];
Cio3 -> 'AO' + 'HRP2' [1];

//		R 2:

metabolite Cfa2 hide, Cia2 hide, Cib2 hide;
'ADH' + 'NADH' -> Cia2 [6.72222e-007];
Cia2 -> 'ADH' + 'NADH' [3.3e-006];
Cia2 + 'acetone' -> Cib2 [0.077];
Cib2 -> Cia2 + 'acetone' [7e-006];
Cib2 -> Cfa2 + 'NAD' [7e-005];
Cfa2 -> 'ADH' + 'isopropanol' [3.3e-005];

//		R 1:

metabolite Cfa1 hide, Cia1 hide, Cib1 hide;
'G_1DH' + 'NAD' -> Cia1 [0.127907];
Cia1 -> 'G_1DH' + 'NAD' [0.004];
Cia1 + 'glucose' -> Cib1 [0.0006875];
Cib1 -> Cia1 + 'glucose' [0.002];
Cib1 -> Cfa1 + 'NADH' [0.02];
Cfa1 -> 'G_1DH' + 'gluconolacrone' [0.04];
```

3. LacOH batch mode

```
title = "LacOH_batch";
geometry = 1000:1000;

metabolite
		'EtOH',
		'acetaldehyde',
		'NADH',
		'Lactate',
		'H2O2',
		'LO',
		'Pyruvate',
		'ABTS',
		'ABTSox',
		'POD',
		'NAD',
		'ADH';

//		R 4:

metabolite Cia4 hide;
'ABTSox' + 'NADH' -> Cia4 [1e-004];
Cia4 -> 'ABTS' + 'NAD' [0.01];

//		R 3:

metabolite Cfa3 hide, Cfb3 hide, Cia3 hide, Cib3 hide;
'POD' + 'H2O2' -> Cia3 [41.8];
Cia3 -> 'POD' + 'H2O2' [0.0076];
'POD' + 'ABTS' -> Cib3 [1.16111];
Cib3 -> 'POD' + 'ABTS' [0.0076];
Cia3 + 'ABTS' -> Cfa3 [1.16111];
Cib3 + 'H2O2' -> Cfb3 [41.8];
Cfa3 -> Cia3 + 'ABTS' [0.0076];
```

```
Cfb3 -> Cib3 + 'H2O2' [0.0076];
Cfa3 -> 'POD' + 'ABTSox' [0.076];
Cfb3 -> 'POD' + 'ABTSox' [0.076];

//		R 2:

metabolite Cf2 hide;
'LO' + 'Lactate' -> Cf2 [0.327662];
Cf2 -> 'LO' + 'Lactate' [0.002383];
metabolite Cio2 hide;
Cf2 -> Cio2 + 'H2O2' [0.02383];
Cio2 -> 'LO' + 'Pyruvate' [1];

//		R 1:

metabolite Cfa1 hide, Cfb1 hide, Cia1 hide, Cib1 hide;
'ADH' + 'EtOH' -> Cia1 [0.0344309];
Cia1 -> 'ADH' + 'EtOH' [0.00308];
'ADH' + 'NAD' -> Cib1 [8.47];
Cib1 -> 'ADH' + 'NAD' [0.00308];
Cia1 + 'NAD' -> Cfa1 [8.47];
Cib1 + 'EtOH' -> Cfb1 [0.0344309];
Cfa1 -> Cia1 + 'NAD' [0.00308];
Cfb1 -> Cib1 + 'EtOH' [0.00308];
metabolite Cio1 hide;
Cfa1 -> Cio1 + 'acetaldehyde' [0.0308];
Cfb1 -> Cio1 + 'acetaldehyde' [0.0308];
Cio1 -> 'ADH' + 'NADH' [1];

init (20 mM, 'EtOH');
init (500 uM, 'Lactate');
init (1.12uM, 'LO');
init (100uM, 'ABTS');
init (0.00347uM, 'POD');
init (250 uM, 'NAD');
init (14.06 uM, 'ADH');
```

4. LacOH protosensor mode:

```
title = "LacOH_protosensor";
geometry = 1500:1500;

metabolite
		'EtOH',
		'acetaldehyde',
		'NADH',
		'Lactate',
		'H2O2',
		'LO',
		'Pyruvate',
		'ABTS',
		'ABTSox',
		'POD',
		'NAD',
		'ADH';

compartment {
		geometry = 1000:1000+0+0+0;		// 10000 nm long, 10000 nm diameter
		init (22.4uM, 'LO');
		init (5000uM, 'NAD');
		init (2000 uM, 'ABTS');
		init (0.0694uM, 'POD');
		init (281.2uM, 'ADH');
}

diffusion (Lactate) = 5e-3; // Arrayed lipid bilayer chambers allow single-molecule analysis of membrane transporter activity
diffusion (EtOH) = 1e-2; // Permeable ~ethanol/H2O, Molecular Biology of the Cell. 4th edition.

//		R 4:
```

```
metabolite Cia4 hide;
'ABTSox' + 'NADH' -> Cia4 [1e-004];
Cia4 -> 'ABTS' + 'NAD' [0.01];

//        R 3:

metabolite Cfa3 hide, Cfb3 hide, Cia3 hide, Cib3 hide;
'POD' + 'H2O2' -> Cia3 [41.8];
Cia3 -> 'POD' + 'H2O2' [0.0076];
'POD' + 'ABTS' -> Cib3 [1.16111];
Cib3 -> 'POD' + 'ABTS' [0.0076];
Cia3 + 'ABTS' -> Cfa3 [1.16111];
Cib3 + 'H2O2' -> Cfb3 [41.8];
Cfa3 -> Cia3 + 'ABTS' [0.0076];
Cfb3 -> Cib3 + 'H2O2' [0.0076];
Cfa3 -> 'POD' + 'ABTSox' [0.076];
Cfb3 -> 'POD' + 'ABTSox' [0.076];

//        R 2:

metabolite Cf2 hide;
'LO' + 'Lactate' -> Cf2 [0.327662];
Cf2 -> 'LO' + 'Lactate' [0.002383];
metabolite Cio2 hide;
Cf2 -> Cio2 + 'H2O2' [0.02383];
Cio2 -> 'LO' + 'Pyruvate' [1];

//        R 1:

metabolite Cfa1 hide, Cfb1 hide, Cia1 hide, Cib1 hide;
'ADH' + 'EtOH' -> Cia1 [0.0344309];
Cia1 -> 'ADH' + 'EtOH' [0.00308];
'ADH' + 'NAD' -> Cib1 [8.47];
Cib1 -> 'ADH' + 'NAD' [0.00308];
Cia1 + 'NAD' -> Cfa1 [8.47];
Cib1 + 'EtOH' -> Cfb1 [0.0344309];
Cfa1 -> Cia1 + 'NAD' [0.00308];
Cfb1 -> Cib1 + 'EtOH' [0.00308];
metabolite Cio1 hide;
Cfa1 -> Cio1 + 'acetaldehyde' [0.0308];
Cfb1 -> Cio1 + 'acetaldehyde' [0.0308];
Cio1 -> 'ADH' + 'NADH' [1];

init (0 mM, 'EtOH');
init (500 uM, 'Lactate');
```

5. GluNOx batch mode:

HSIM model code:

```
title = "GluNOx_batch";
geometry = 1000:1000;

metabolite
        'glucose',
        'gluconolactone',
        'G_1DH',
        'NO3',
        'NO2',
        'NR',
        'NADH',
        'NAD',
        'N2O3',
        'DAFF',
        'NO2b',
        'O2',
        'NO',
        'DAF';
```

```
//	R 8: NO decay (Application of carbon fiber composite minielectrodes for measurement of kinetic constants of nitric oxide decay in solution.)

metabolite Cf8 hide;
'NO' -> Cf8 [1.9e-007];

//	R 7:

metabolite Cf7 hide;
'NO2b' + 'NO' -> Cf7 [0.25e-005];
Cf7 -> 'NO2b' + 'NO'  [6.25e-007];
Cf7 -> 'N2O3' [0.2];

//	R 6:

metabolite Cf6 hide;
'O2' + 'NO' -> Cf6 [0.25e-005];
Cf6 -> 'O2' + 'NO'  [6.25e-007];
Cf6 -> 'NO2b' [0.0002];

//	R 5:

metabolite Cf5 hide;
'DAF' + 'N2O3' -> Cf5 [0.25e-005];
Cf5 -> 'DAF' + 'N2O3'  [6.25e-007];
Cf5 -> 'DAFF' [0.2];

// metabolite Cf5 hide;
// 'DAF' + 'NO' -> Cf5 [0.25e-005];
// Cf5 -> 'DAF' + 'NO'  [6.25e-007];
// Cf5 -> 'DAFF' [0.000628];

//	R 2:

metabolite Cfa2 hide, Cfb2 hide, Cia2 hide, Cib2 hide;
'NR' + 'NO3' -> Cia2 [0.385];
Cia2 -> 'NR' + 'NO3' [0.00021];
'NR' + 'NADH' -> Cib2 [0.144375];
Cib2 -> 'NR' + 'NADH' [0.000021];
Cia2 + 'NADH' -> Cfa2 [0.144375];
Cib2 + 'NO3' -> Cfb2 [0.385];
Cfa2 -> Cia2 + 'NADH' [0.000021];
Cfb2 -> Cib2 + 'NO3' [0.00021];
metabolite Cio2 hide;
Cfa2 -> Cio2 + 'NO2' [0.021];
Cfb2 -> Cio2 + 'NO2' [0.021];
Cio2 -> 'NR' + 'NAD' [1];

//	R 3:

metabolite Cfa3 hide, Cfb3 hide, Cib3 hide;
'NR' + 'NO2' -> Cib3 [0.0743243];
Cib3 -> 'NR' + 'NO2' [2e-005];
Cib3 + 'NADH' -> Cfa3 [0.1375];
Cib2 + 'NO2' -> Cfb3 [0.0743243];
Cfa3 -> Cib3 + 'NADH' [2e-005];
Cfb3 -> Cib2 + 'NO2' [2e-005];
metabolite Cio3 hide;
Cfa3 -> Cio3 + 'NO' [0.0002];
Cfb3 -> Cio3 + 'NO' [0.0002];
Cio3 -> 'NR' + 'NAD' [1];

//	R 1:

metabolite Cfa1 hide, Cia1 hide, Cib1 hide;
'G_1DH' + 'NAD' -> Cia1 [0.023913];
Cia1 -> 'G_1DH' + 'NAD' [0.004];
Cia1 + 'glucose' -> Cib1 [0.0006875];
Cib1 -> Cia1 + 'glucose' [0.002];
```

```
Cib1 -> Cfa1 + 'NADH' [0.02];
Cfa1 -> 'G_1DH' + 'gluconolactone' [0.04];

init (5000uM, 'glucose');
init (2uM, 'G_1DH');
init (5000uM, 'NO3');
init (20uM, 'NO2');
init (100uM, 'NAD');
init (0.253mM, 'O2');
init (10uM, 'DAF');
init (4.2uM, 'NR');
```

6. GluNOx protosensor mode

```
title = "GluNOx_protosensor";
geometry = 1500:1500;

metabolite
        'glucose',
        'gluconolactone',
        'G_1DH',
        'NO3',
        'NO2',
        'NR',
        'NADH',
        'NAD',
        'N2O3',
        'DAFF',
        'NO2b',
        'O2',
        'NO',
        'DAF';

compartment {
        geometry = 1000:1000+0+0+0;     // 10000 nm long, 10000 nm diameter
        init (115.092uM, 'G_1DH');
        init (2000uM, 'NAD');
        init (200uM, 'DAF');
        init (84uM, 'NR');
}

diffusion (glucose) = 5e-3; // Arrayed lipid bilayer chambers allow single-molecule analysis of membrane transporter activity
diffusion (NO3) = 5e-3; //
diffusion (O2) = 5e-3; //

//      R 8: NO decay (Application of carbon fiber composite minielectrodes for measurement of kinetic constants of nitric oxide decay in solution.)

metabolite Cf8 hide;
'NO' -> Cf8 [1.9e-007];

//      R 7:

metabolite Cf7 hide;
'NO2b' + 'NO' -> Cf7 [0.25e-005];
Cf7 -> 'NO2b' + 'NO'  [6.25e-007];
Cf7 -> 'N2O3' [0.2];

//      R 6:

metabolite Cf6 hide;
'O2' + 'NO' -> Cf6 [0.25e-005];
Cf6 -> 'O2' + 'NO'  [6.25e-007];
Cf6 -> 'NO2b' [0.0002];

//      R 5:

metabolite Cf5 hide;
'DAF' + 'N2O3' -> Cf5 [0.25e-005];
```

```
Cf5 -> 'DAF' + 'N2O3'  [6.25e-007];
Cf5 -> 'DAFF' [0.2];

// metabolite Cf5 hide;
// 'DAF' + 'NO' -> Cf5 [0.25e-005];
// Cf5 -> 'DAF' + 'NO'  [6.25e-007];
// Cf5 -> 'DAFF' [0.000628];

//		R 2:

metabolite Cfa2 hide, Cfb2 hide, Cia2 hide, Cib2 hide;
'NR' + 'NO3' -> Cia2 [0.385];
Cia2 -> 'NR' + 'NO3' [0.00021];
'NR' + 'NADH' -> Cib2 [0.144375];
Cib2 -> 'NR' + 'NADH' [0.000021];
Cia2 + 'NADH' -> Cfa2 [0.144375];
Cib2 + 'NO3' -> Cfb2 [0.385];
Cfa2 -> Cia2 + 'NADH' [0.000021];
Cfb2 -> Cib2 + 'NO3' [0.00021];
metabolite Cio2 hide;
Cfa2 -> Cio2 + 'NO2' [0.021];
Cfb2 -> Cio2 + 'NO2' [0.021];
Cio2 -> 'NR' + 'NAD' [1];

//		R 3:

metabolite Cfa3 hide, Cfb3 hide, Cib3 hide;
'NR' + 'NO2' -> Cib3 [0.0743243];
Cib3 -> 'NR' + 'NO2' [2e-005];
Cib3 + 'NADH' -> Cfa3 [0.1375];
Cib2 + 'NO2' -> Cfb3 [0.0743243];
Cfa3 -> Cib3 + 'NADH' [2e-005];
Cfb3 -> Cib2 + 'NO2' [2e-005];
metabolite Cio3 hide;
Cfa3 -> Cio3 + 'NO' [0.0002];
Cfb3 -> Cio3 + 'NO' [0.0002];
Cio3 -> 'NR' + 'NAD' [1];

//		R 1:

metabolite Cfa1 hide, Cia1 hide, Cib1 hide;
'G_1DH' + 'NAD' -> Cia1 [0.023913];
Cia1 -> 'G_1DH' + 'NAD' [0.004];
Cia1 + 'glucose' -> Cib1 [0.0006875];
Cib1 -> Cia1 + 'glucose' [0.002];
Cib1 -> Cfa1 + 'NADH' [0.02];
Cfa1 -> 'G_1DH' + 'gluconolactone' [0.04];

init (5000uM, 'glucose');
init (5000uM, 'NO3');
init (0uM, 'NO2');
init (0.253mM, 'O2');
```

IV. Example of BIOCHAM code for GluNOx

BIOCHAM code for the three synthetic circuits described in this study can be found attached as .bc notebooks (**Computer Code EV5,6,7)**, executable one the BIOCHAM Online server, or after downloading the binaries. Below is a complete example of the GluNOx protosensor circuit (**Computer Code EV7**).

BIOCHAM 3.7.4

Copyright (C) 2003-2017 INRIA, EPI Lifeware, Paris-Rocquencourt, France,
license GNU GPL 2, http://lifeware.inria.fr/biocham/

```
In [0]: parameter (k1, 0.0019).

In [1]: parameter (k2, 7.77313e-006).

In [2]: parameter (k3, 0.2).

In [3]: parameter (k4, 2).

In [4]: parameter (k5, 0.00777313).

In [5]: parameter (k6, 200).

In [6]: parameter (k7, 2000).

In [7]: parameter (k8, 0.00777313).

In [8]: parameter (k9, 200).

In [10]: parameter (k11, 1.05042e-006).

In [11]: parameter (k12, 0.2).

In [12]: parameter (k13, 1.94328e-006).

In [13]: parameter (k14, 0.2).

In [14]: parameter (k15, 1.94328e-006).

In [15]: parameter (k16, 1.05042e-006).

In [16]: parameter (k17, 0.2).

In [17]: parameter (k18, 0.2).

In [18]: parameter (k19, 2).

In [19]: parameter (k20, 2).

In [20]: parameter (k21, 10000).

In [21]: MA(k1) for NO => volatNO.
MA(k1) for NO=>volatNO

In [22]: MA(k2) for O2 + NO => Cf6.
MA(k2) for O2+NO=>Cf6

In [23]: MA(k3) for Cf6 => O2 + NO.
MA(k3) for Cf6=>O2+NO

In [24]: MA(k4) for Cf6 => O2 + NO2b.
MA(k4) for Cf6=>O2+NO2b

In [25]: MA(k5) for NO + NO2b => Cf5.
MA(k5) for NO+NO2b=>Cf5

In [26]: MA(k6) for Cf5 => NO + NO2b.
MA(k6) for Cf5=>NO+NO2b

In [27]: MA(k7) for Cf5 => N2O3.
MA(k7) for Cf5=>N2O3

In [28]: MA(k8) for DAF + N2O3 => Cf4.
MA(k8) for DAF+N2O3=>Cf4
```

```
In [29]: MA(k9) for Cf4 => DAF + N2O3.
```
MA(k9) for Cf4=>DAF+N2O3

```
In [30]: MA(k10) for Cf4 => DAFF.
```
MA(k10) for Cf4=>DAFF

```
In [31]: MA(k11) for NR + NO2 => Cia3.
```
MA(k11) for NR+NO2=>Cia3

```
In [32]: MA(k12) for Cia3 => NR + NO2.
```
MA(k12) for Cia3=>NR+NO2

```
In [33]: MA(k13) for NR + NADH => Cib3.
```
MA(k13) for NR+NADH=>Cib3

```
In [34]: MA(k14) for Cib3 => NR + NADH.
```
MA(k14) for Cib3=>NR+NADH

```
In [35]: MA(k15) for Cia3 + NADH => Cfa3.
```
MA(k15) for Cia3+NADH=>Cfa3

```
In [36]: MA(k16) for Cib3 + NO2 => Cfb3.
```
MA(k16) for Cib3+NO2=>Cfb3

```
In [37]: MA(k17) for Cfa3 => Cia3 + NADH.
```
MA(k17) for Cfa3=>Cia3+NADH

```
In [38]: MA(k18) for Cfb3 => Cib3 + NO2.
```
MA(k18) for Cfb3=>Cib3+NO2

```
In [39]: MA(k19) for Cfa3 => Cio3 + NO.
```
MA(k19) for Cfa3=>Cio3+NO

```
In [40]: MA(k20) for Cfb3 => Cio3 + NO.
```
MA(k20) for Cfb3=>Cio3+NO

```
In [41]: MA(k21) for Cio3 => NR + NAD.
```
MA(k21) for Cio3=>NR+NAD

```
In [42]: parameter (k22, 5.44119e-005).
```

```
In [43]: parameter (k23, 21).
```

```
In [44]: parameter (k25, 0.000204045).
```

```
In [45]: parameter (k26, 5.44119e-005).
```

```
In [46]: parameter (k27, 21).
```

```
In [47]: parameter (k28, 21).
```

```
In [48]: parameter (k29, 210).
```

```
In [49]: parameter (k30, 210).
```

```
In [50]: parameter (k31, 10000).
```

```
In [51]: parameter (k32, 3.37961e-007).
```

```
In [52]: parameter (k33, 40).
```

```
In [53]: parameter (k34, 9.71641e-009).
```

```
In [54]: parameter (k35, 20).
```

```
In [55]: parameter (k36, 200).
```

```
In [56]: parameter (k37, 400).
```

```
In [57]: MA(k22) for NR + NO3 => Cia2.
```
MA(k22) for NR+NO3=>Cia2

```
In [58]: MA(k23) for Cia2 => NR + NO3.
```
MA(k23) for Cia2=>NR+NO3

```
In [59]: MA(k25) for Cia2 + NADH => Cfa2.
```
MA(k25) for Cia2+NADH=>Cfa2

```
In [60]: MA(k26) for Cib3 + NO3 => Cfb2.
```
MA(k26) for Cib3+NO3=>Cfb2

```
In [61]: MA(k27) for Cfa2 => Cia2 + NADH.
```
MA(k27) for Cfa2=>Cia2+NADH

```
In [62]: MA(k28) for Cfb2 => Cib3 + NO3.
```
MA(k28) for Cfb2=>Cib3+NO3

```
In [63]: MA(k29) for Cfa2 => Cio2 + NO2.
```
MA(k29) for Cfa2=>Cio2+NO2

```
In [64]: MA(k30) for Cfb2 => Cio2 + NO2.
```
MA(k30) for Cfb2=>Cio2+NO2

```
In [65]: MA(k31) for Cio2 => NR + NAD.
```
MA(k31) for Cio2=>NR+NAD

```
In [66]: MA(k32) for G_1DH + NAD => Cia1.
```
MA(k32) for G_1DH+NAD=>Cia1

```
In [67]: MA(k33) for Cia1 => G_1DH + NAD.
```
MA(k33) for Cia1=>G_1DH+NAD

```
In [68]: MA(k34) for Cia1 + glucose => Cib1.
```
MA(k34) for Cia1+glucose=>Cib1

```
In [69]: MA(k35) for Cib1 => Cia1 + glucose.
```
MA(k35) for Cib1=>Cia1+glucose

```
In [70]: MA(k36) for Cib1 => Cfa1 + NADH.
```
MA(k36) for Cib1=>Cfa1+NADH

```
In [71]: MA(k37) for Cfa1 => G_1DH + gluconolacrone.
```
MA(k37) for Cfa1=>G_1DH+gluconolacrone

```
In [72]: parameter (k38, 5e-3).
```

```
In [73]: parameter (k39, 5e-3).
```

```
In [74]: parameter (k40, 5e-3).
```

```
In [75]: MA(k38) for glucoseext => glucose.
```
MA(k38) for glucoseext=>glucose

```
In [76]: MA(k39) for NO3ext => NO3.
```

MA(k39) for NO3ext=>NO3

```
In [77]: MA(k40) for NO2ext => NO2.
```

MA(k40) for NO2ext=>NO2

```
In [78]: present(glucose, 0).
```

```
In [79]: present(gluconolacrone, 0).
```

```
In [80]: present(G_1DH, a).
```

```
In [81]: present(NO3, 0).
```

```
In [82]: present(NO2, 0).
```

```
In [83]: present(NR, b).
```

```
In [84]: present(NADH, 0).
```

```
In [85]: present(NAD, g).
```

```
In [86]: present(N2O3, 0).
```

```
In [87]: present(DAFF, 0).
```

```
In [88]: present(NO2b, 0).
```

```
In [89]: present(O2, 79780608).
```

```
In [90]: present(NO, 0).
```

```
In [91]: present(DAF, 63067600).
```

```
In [92]: present(volatNO, 0).
```

```
In [93]: present(NOp, 0).
```

```
In [94]: present(Cf6, 0).
```

```
In [95]: present(Cf5, 0).
```

```
In [96]: present(Cf4, 0).
```

```
In [97]: present(Cfa3, 0).
```

```
In [98]: present(Cfb3, 0).
```

```
In [99]: present(Cia3, 0).
```

```
In [100]: present(Cib3, 0).
```

```
In [101]: present(Cio3, 0).
```

```
In [102]: present(Cfa2, 0).
```

```
In [103]: present(Cia2, 0).
```

```
In [104]: present(Cfa2, 0).
```

```
In [105]: present(Cfb2, 0).
```

```
In [106]: present(Cia2, 0).
```

```
In [107]: present(Cio2, 0).
```

```
In [108]: present(Cfa1, 0).
```

```
In [109]: present(Cia1, 0).
```

```
In [110]: parameter(b, 26488392).
```

```
In [111]: parameter(a, 36295404).
```

```
In [112]: parameter(g, 630676000).
```

```
In [113]: hide_molecules(?).
```

```
In [114]: show_molecules({NADH, DAFF}).
```

```
In [115]: present(glucoseext, d).
```

```
In [116]: present(NO3ext, e).
```

```
In [117]: present(NO2ext, f).
```

```
In [118]: parameter(d, 438319820).
```

```
In [119]: parameter(e, 315338000).
```

```
In [120]: parameter(f, 1261356).
```

```
In [121]: numerical_simulation(3600).
```

```
Simulation time: 21.773s
```

```
In [122]: plot.
```

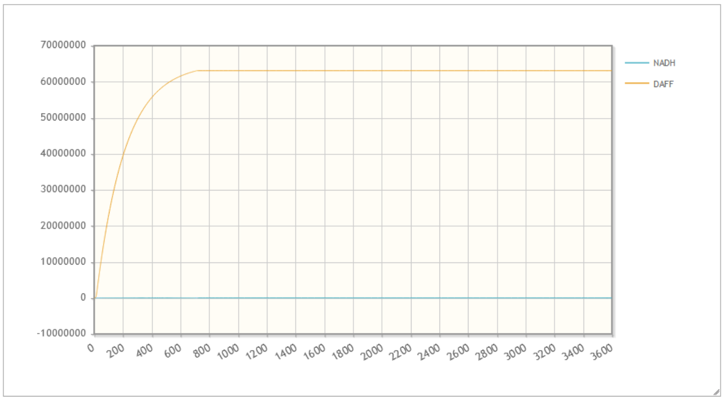


```
In [123]: validity_domain(F(G((Time>T) & (N > [NADH]) & ([DAFF] > R)))).
```

```
T < 3609.51, N > 0.000219875, R < 6.30676e+07
Time elapsed : 408 ms
```

```
In [124]: sensitivity([g,a,b],[0.5,0.5,0.5],F(G((N > [NADH]) & ([DAFF] > R))), [N,R], [1000,1000000], 300).
```

```
g=9.46014e+08 violation 2301.92 (0.15s)
 g=3.15338e+08 violation 2376.75 (0.14s)
Sensitivity[g] 9.33592e-11

 a=5.44431e+07 violation 2313.69 (0.15s)
 a=1.81477e+07 violation 2513.63 (0.13s)
Sensitivity[a] 5.89989e-10

 b=3.97326e+07 violation 1246.94 (0.16s)
 b=1.32442e+07 violation 5774.58 (0.14s)
Sensitivity[b] 1.97303e-07
```

In [125]:
```
landscape([g,a],[(0,1000000000),(0,100000000)],F(G((N > [NADH]) & ([DAFF] > R))), [N,R], [3500,50000000], 10, 300, landG1DHADH).
```

In [127]:
```
search_parameters_cmaes([g,a,b],[(0,1000000000),(0,100000000),(0,100000000)],F(G((N > [NADH]) & ([DAFF] > R))), [N,R], [3500,50000000],300).
```

```
## Exit function optimize (Thu Nov 16 09:45:15 2017) ##
Best parameter coefs : 1.04272 1.15752 0.957613
Found Parameters :
parameter(g,657619411).
parameter(a,42012496.1).
parameter(b,25365634.1).
Time elapsed 219.46 s
```

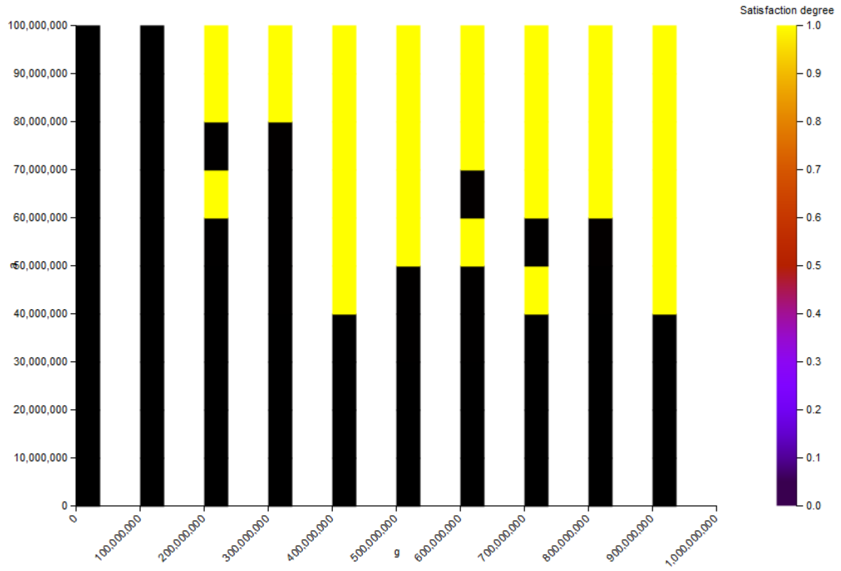

Supplement: Supplementary file 1 — Appendix [file MSB-14-e7845-s001.pdf]
